# Dissecting the Satellite DNA Landscape in Three Cactophilic *Drosophila* Sequenced Genomes

**DOI:** 10.1534/g3.117.042093

**Published:** 2017-06-28

**Authors:** Leonardo G. de Lima, Marta Svartman, Gustavo C. S. Kuhn

**Affiliations:** Laboratório de Citogenômica Evolutiva, Departamento de Biologia Geral, Instituto de Ciências Biológicas, Universidade Federal de Minas Gerais, Belo Horizonte, Minas Gerais 31270-901, Brazil

**Keywords:** satellite DNA, cactophilic *Drosophila*, centromeres, telomeres, concerted evolution

## Abstract

Eukaryote genomes are replete with repetitive DNAs. This class includes tandemly repeated satellite DNAs (satDNA) which are among the most abundant, fast evolving (yet poorly studied) genomic components. Here, we used high-throughput sequencing data from three cactophilic *Drosophila* species, *D. buzzatii*, *D. seriema*, and *D. mojavensis*, to access and study their whole satDNA landscape. In total, the *RepeatExplorer* software identified five satDNAs, three previously described (*pBuM*, *DBC-150* and *CDSTR198*) and two novel ones (*CDSTR138* and *CDSTR130*). Only *pBuM* is shared among all three species. The satDNA repeat length falls within only two classes, between 130 and 200 bp or between 340 and 390 bp. FISH on metaphase and polytene chromosomes revealed the presence of satDNA arrays in at least one of the following genomic compartments: centromeric, telomeric, subtelomeric, or dispersed along euchromatin. The chromosomal distribution ranges from a single chromosome to almost all chromosomes of the complement. Fiber-FISH and sequence analysis of contigs revealed interspersion between *pBuM* and *CDSTR130* in the microchromosomes of *D. mojavensis*. Phylogenetic analyses showed that the pBuM satDNA underwent concerted evolution at both interspecific and intraspecific levels. Based on RNA-seq data, we found transcription activity for *pBuM* (in *D. mojavensis*) and *CDSTR198* (in *D. buzzatii*) in all five analyzed developmental stages, most notably in pupae and adult males. Our data revealed that cactophilic *Drosophila* present the lowest amount of satDNAs (1.9–2.9%) within the *Drosophila* genus reported so far. We discuss how our findings on the satDNA location, abundance, organization, and transcription activity may be related to functional aspects.

The genomes of many organisms are replete with highly repetitive (>1000 copies) tandemly repeated DNA sequences, commonly known as satellite DNAs (satDNAs) (Tautz 1993). Long and homogeneous arrays made of satDNA repeats are located in the heterochromatin (Charlesworth *et al.* 1994; Plohl 2012; Beridze 2013; Khost *et al.* 2017), but recent studies also revealed the presence of short arrays dispersed along the euchromatin (Brajković *et al.* 2012; Kuhn *et al.* 2012; Larracuente 2014; Pavlek *et al.* 2015). SatDNAs do not have the ability to transpose by themselves as transposable elements (TEs) do. However, there are some reported examples showing that TEs may act as a substrate for satDNA emergence and mobility (Dias *et al.* 2015; Meštrović *et al.* 2015; Satović *et al.* 2016).

The whole collection of satDNAs makes up large portions (usually >30%) of animal and plant genomes (reviewed by Plohl *et al.* (2014)). Although satDNAs do not code for proteins, they may play important cellular roles, including participation in chromatin packaging (Blattes *et al.* 2006; Feliciello *et al.* 2015), centromere formation/maintenance (Rošić *et al.* 2014; Aldrup-MacDonald *et al.* 2016), and gene regulation (Menon *et al.* 2014; Feliciello *et al.* 2015; Urrego *et al.* 2017).

Despite their abundance, diversity and contribution to genomic architecture and function, our knowledge about several features of satDNAs is still limited. In the past decades, satDNAs have been mostly studied from a small sample of cloned repeats obtained by biased experimental approaches (usually by restriction digestion and/or PCR), isolated from one or few species. Experimental strategies for the identification of satDNAs were expensive, time-consuming, and insufficient for the identification of the whole collection of satDNAs from any chosen genome.

Next-generation sequencing technologies have provided a revolution in the number of species with sequenced genomes, while new and efficient bioinformatic tools have been specifically developed toward genome-wide identification of repetitive DNAs. Consequently, we now have new tools and strategies to access the whole collection of satDNAs from a given genome. For example, software tools known as *RepeatExplorer* have been successfully used for genome-wide characterization of repetitive DNAs from several animal and plant genomes, including those sequenced with >1× coverage (Barghini *et al.* 2014; Marques *et al.* 2015; Ruiz-Ruano *et al.* 2016; Zhang *et al.* 2017). This algorithm directly uses short next-generation sequencing reads as rough material for the identification of repeats. Together with the results from similarity searches and abundance, the repeat families can be identified and classified.

Within the genus *Drosophila*, most studies on satDNA were conducted in *D. melanogaster* and in a few closely related species from the *melanogaster* group (*e.g.*, Strachan *et al.* 1985; Kuhn *et al.* 2012; Larracuente 2014; Jagannathan *et al.* 2017). The study of satDNAs of species distantly related to *D. melanogaster* are expected to broaden the understanding of this major fraction of the eukaryote genome. In this context, the *repleta* group is of particular interest. It contains at least 100 species that breed in cactuses in North and South America (Oliveira *et al.* 2012). Species from the *repleta* group are separated from the *melanogaster* group by >40 MY (Powell 1997). Intense vertical studies in some species of this group revealed several aspects related to chromosome and genome evolution that have broad interest (*e.g.*, Cáceres *et al.* 1999; Negre *et al.* 2005; Kuhn *et al.* 2009; Guillén *et al.* 2015).

At present, three *repleta* group species have available sequenced genomes: *D. mojavensis* (Drosophila 12 Genomes Consortium 2007), *D. buzzatii* (Guillén *et al.* 2015), and *D. seriema* (Dias G.B., M. Svartman and G.C.S. Kuhn, unpublished data). *D*. *buzzatii* and *D. seriema* belong to the *buzzatii* cluster, a monophyletic group of South American origin that contains seven species morphologically very similar and came from a radiation process dated at 6 MYA (Manfrin and Sene 2006; Oliveira *et al.* 2012). *D. mojavensis* lives in the deserts and dry tropical forests of the southwestern United States and Mexico (Reed *et al.* 2007). The time since the split between *D. buzzatii* and *D. mojavensis* has been estimated at 11 MYA (Oliveira *et al.* 2012; Guillén *et al.* 2015).

Previous studies in *D. buzzatii* and *D. seriema* conducted before the genomic era allowed the identification of three satDNA families. The first family, named *pBuM*, can be divided into two subfamilies according to its primary structure and size of the repeat units (Kuhn and Sene 2005). The *pBuM*-1 subfamily is comprised of *alpha* repeat units of ∼190 bp, whereas the *pBuM*-2 subfamily consists of 370-bp composite repeat units called *alpha/beta*, each one consisting of an *alpha* (∼190 bp) followed by a *beta* sequence (∼180 bp) of unknown origin. DNA hybridization data revealed pBuM-1 to be the major repeat variant present in *D. buzzatii* but pBuM-2 as the major repeat variant in *D. seriema*.

The second family, named *DBC-150*, consists of 150-bp long repeat units. This family is abundant in *D. seriema* but virtually absent in *D. buzzatii* (Kuhn *et al.* 2007). Finally, the third satDNA family, named *SSS13*9, with 139-bp-long repeat units is abundant in *D. seriema* but absent in *D. buzzatii* (Franco *et al.* 2008). There is no significant sequence similarity among *pBuM*, DBC-150, and *SSS139* satDNA repeats, suggesting that these families have independent evolutionary origins.

Three sequencing platforms (Sanger, 454, and Illumina) (Guillén *et al.* 2015) have been used to sequence the *D. buzzatii* genome, which became publicly available in 2015 (http://dbuz.uab.cat). In a preliminary approach, we used the Tandem Repeats Finder (TRF) software (version 4.04) (Benson 1999) to search for satDNAs with repeats longer than 50 bp in the *D. buzzatii* contigs. The two most abundant tandem repeat families identified were *pBuM*-1 (*alpha* repeats) and a novel family that we named *CDSTR198*, with 198-bp-long repeat units (Guillén *et al.* 2015). However, in *D. melanogaster* and *D. virilis*, for example, several abundant satDNA families showed repeat units <10-bp long (Gall *et al.* 1971; Lohe *et al.* 1993). Therefore, a new satDNA screen is necessary in the *D. buzzatii* sequenced genome in order to look for the presence of small-size satDNA repeat motifs.

There are no detailed studies involving satDNAs in *D. mojavensis*. Melters *et al.* (2013) developed a bioinformatic pipeline to identify the most abundant tandem repeats from 282 selected sequenced genomes from animal and plant species, including some *Drosophila* species. A satDNA with 183-bp-long repeat units was identified as the most abundant satDNA of *D. mojavensis*. Most recently, we showed that this satDNA actually belongs to the *pBuM*-1 satDNA subfamily (*alpha* repeats), previously described in *D. buzzatii* (Guillén *et al.* 2015).

Our group has recently sequenced the genome of *D. seriema* using the MiSeq platform (Dias *et al.*, unpublished data). The availability of three sequenced genomes (*D. buzzatii*, *D. seriema*, and *D. mojavensis*) provides an unprecedented opportunity to study the satDNA collection from each species and to compare them in a scale never possible before. We combined bioinformatic, phylogenetic, and molecular cytogenetic tools to study the satDNA fraction from these three cactophilic *Drosophila* species. The resulting data are discussed in the context of satDNA genomic distribution, evolution, and potential functional roles.

## Materials and Methods

### Genomic data

The Illumina sequence reads from *D. buzzatii*, *D. mojavensis*, and *D. seriema* used for identification of satDNAs were obtained from three different sources. *D. buzzatii* reads (76× coverage) were generated by the Prof. Alfredo Ruiz group at Universitat Autònoma de Barcelona and were used for the genome assembly of *D. buzzatii* (Guillén *et al.* 2015). All *D. buzzatii* Illumina reads used on this paper were downloaded directly from the *Drosophila buzzatii* genome project webpage (http://dbuz.uab.cat). These data are publicly available for download on the FTP section: http://dbuz.uab.cat/ftp.php. We used *D. mojavensis* (SRX2932915) sequence reads (20× coverage) generated by Prof. Bernardo de Carvalho (Universidade Federal do Rio de Janeiro, Brazil), and *D. seriema* (ERX2037878) sequence reads (20× coverage) were generated by our group (Dias *et al.*, unpublished data).

### Identification of satellite DNAs

Similarity-based clustering, repeat identification, and classification were performed using *RepeatExplorer* (Novák *et al.* 2013) with whole-genome shotgun *Illumina* reads from *D. buzzatii*, *D. mojavensis*, and *D. seriema*. Initially, files containing all sequence reads from each species were uploaded (trimmed at 100 bp). The clustering analysis used *RepeatExplorer* default parameters. Clusters containing possible tandemly repeated satDNA families were identified based on the resultant graph-based clustering and then manually checked for the presence of tandem repeats using the TRF software (version 4.04) (Benson 1999). Genomic proportion was calculated from the number of reads present in each cluster divided by the total number of reads. We searched for clusters with high graph density, which is a typical characteristic of satDNAs families (Novák *et al.* 2013). The *Dotlet* software (Junier and Pagni 2000) was also used to generate a scrutinized description of full length copies of each satDNA family.

### Sequence and phylogenetic analysis

Multiple satDNA sequences were aligned with the *Muscle* algorithm (Edgar 2004) of the MEGA5.05 software (Tamura *et al.* 2011), with manual optimization when necessary. MEGA5.05 was also used for the analysis of nucleotide composition and variability. Phylogenetic trees were constructed with the Neighbor Joining algorithm (Saitou and Nei 1987) of the MEGA program 5:05 (Tamura *et al.* 2011). The genetic distance between sequences was calculated using the “Tamura-Nei model” (Tamura and Nei 1993) after an analysis of best substitution model for the data on MEGA 5.05 (Tamura *et al.* 2011). Statistical evaluation of each branch of the tree was performed using analysis “bootstrap” (1000 replicates).

### Samples, DNA extractions, PCR amplifications, cloning, and sequencing

For our experimental data we used DNA from the same sequenced strains: *D. buzzatii* (strain: ST01), *D. seriema* (strain: D73C3B), and *D. mojavensis* (strain: CI 12 IB -4 g8). DNA extraction of 30–50 adult flies was performed with the Wizard Genomic DNA Purification kit (Promega). PCR reactions consisted of an initial denaturation step of 94° for 3 min, followed by 30 cycles of 94° for 60 sec, 55° for 60 sec, and 72° for 60 sec and then a final extension at 72° for 10 min. The primers used for satDNA amplification are listed in Supplemental Material, Table S1 in File S1. PCR products were excised from 1% agarose gels and purified with the Wizard SV Gel and PCR Clean-up System kit (Promega). After cloning with the pGEM-T-Easy cloning kit (Promega), recombinant plasmids were sequenced on the ABI3130 platform (Myleus Biotechnology).

### In situ hybridization experiments

Chromosome preparations, DNA fibers obtention, single and double-color FISH, and Fiber-FISH experiments were conducted as described in Kuhn *et al.* (2008). The probes labeled with digoxigenin-11-dUTP were detected with antidigoxigenin FITC (Roche) and probes labeled with biotin-14-dATP were detected with NeutrAvidin-rhodamine (Roche). Chromosomes were stained with DAPI (4′, 6-diamidino-2-phenylindole, dihydrochloride salt). The preparations were analyzed under an epifluorescence Zeiss Axiophot 2 microscope equipped with a CCD camera and the images were obtained using the AxioVision software (Zeiss). To determine the size of the DNA fibers, hybridization signals were measured according to the protocol described by Schwarzacher and Heslop-Harrison (2000).

### Transcription analysis

Total RNA-seq data of *D. mojavensis* and *D. buzzatii* (st-1 strain) were those obtained by Guillén *et al.* (2015). Briefly, RNA samples were extracted from 10 to 20 individuals from each of the four development stages (embryos, third-stage larvae, pupae, adult females and males), enriched for mRNA by poly-A tail selection and sequenced by Illumina, generating ∼100 bp reads [see Guillén *et al.* (2015) for details]. All reads were aligned against consensus sequences representing the *pBuM* and *CDSTR198* families from *D. buzzatii* and *pBuM* and *CDSTR*130 from *D. mojavensis* with the Bowtie2 software (Langmead and Salzberg 2012) incorporated into the usegalaxy.org server (Afgan *et al.* 2016). The mapped reads were normalized by the RPKM method (reads per kilobase per million mapped reads; Mortazavi *et al.* 2008).

### Data availability

The authors state that all data necessary for confirming the conclusions presented in the article are represented fully within the article.

## Results and Discussion

### Cactophilic Drosophila repetitive DNAs: general aspects

**Figure 1:**
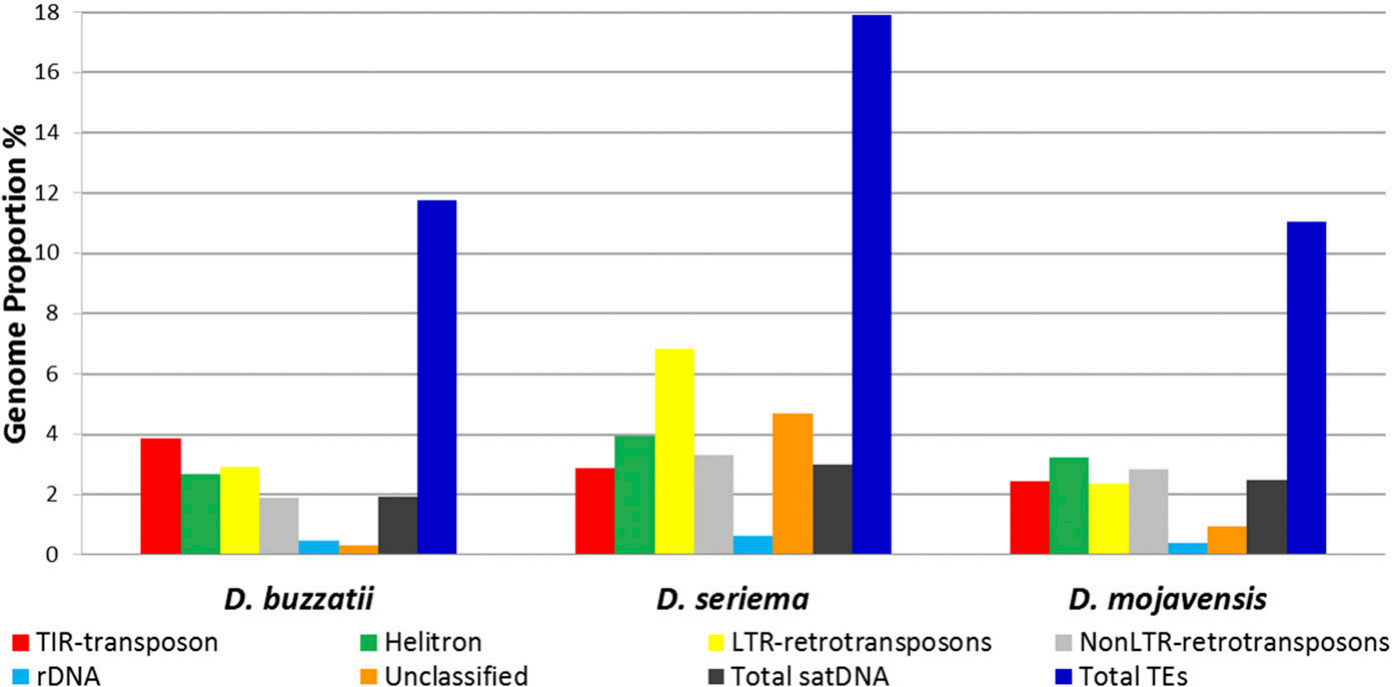
Estimated repetitive DNA abundance in three cactophilic *Drosophila* species.

The *RepeatExplorer* graphic representation containing all identified repetitive DNA clusters in *D. buzzatii*, *D. seriema*, and *D. mojavensis* and their genome proportion (%) is shown in Figures S1–S3 in File S1. Most clusters making >0.01% of the genome could be classified into established groups of repetitive elements, such as TEs, satDNAs, or rDNA sequences (Figure 1 and Tables S2–S4 in File S1).

The satDNA genomic contribution is similar in the three species: ∼1.9% in *D. buzzatii*, ∼2.9% in *D. seriema*, and ∼2.5% in *D. mojavensis*. The genomic contribution of the classified TEs is on average 5.4× higher: ∼12% in *D. buzzatii*, ∼18% in *D. seriema*, and ∼11% in *D. mojavensis*. Rius *et al.* (2016) have recently estimated the TE content of *D. buzzatii* and *D. mojavensis* using the same genomic sequences used in this work, but with a different methodology, and found that TEs represent ∼11% of the *D. buzzatii* and ∼15% of the *D. mojavensis* genomes.

The genomic contribution of the different TE orders [TIR-transposons, Helitrons, long terminal repeat (LTR) retrotransposons, and non-LTR retrotransposons] differs among the three species (Figure 1). TIR-transposons are the most abundant TEs in the *D. buzzatii* genome (3.85%); in *D. seriema*, LTR retrotransposons (6.8%) are the most abundant and in *D. mojavensis*, Helitrons are the most abundant TE elements (3.25%). Conversely, Rius *et al.* (2016) described Helitrons as the most abundant TEs in the *D. buzzatii* and *D. mojavensis* genomes. Interestingly, the genomic contribution of LTR retrotransposons in *D. seriema* (6.8%) is at least two times higher than in *D. buzzatii* (2.9%) or in *D. mojavensis* (2.4%). The contribution of unclassified repetitive elements is also considerably higher in *D. seriema* (18%) than in the other two species (11% and 12%). These results suggest a recent burst of repetitive elements in *D. seriema*.

### Satellite DNA landscape in the three cactophilic Drosophila species

We identified only two previously described satDNA families in *D. buzzatii*. The *pBuM*-1 satDNA (Kuhn and Sene 2005) with 189-bp-long *alpha* repeats is the most abundant, representing 1.7%. The second is *CDSTR198* (Guillén *et al.* 2015), with 198-bp-long repeats and representing 0.2% of the genome. These genomic contributions revealed by *RepeatExplorer* are higher than those obtained by our first contig-based approach, most notably for *pBuM*-1 (0.04% for *pBuM*-1 and 0.03% for *CDSTR198*; Guillén *et al.* 2015). The organization of satDNAs, made of several tandem repeats with high DNA sequence similarity, imposes a huge limitation for assembly computer programs. Consequently, it is very likely that the bulk of *pBuM* and *CDSTR198* satDNA repeats of *D. buzzatii* were omitted from the contigs used in our previous approach. Accordingly, although still low (see discussion below), we consider the values obtained in the present work as the most reliable ones.

We detected four satDNAs in *D. seriema*. The *pBuM*-2 satDNA with ∼340- to 390-bp-long *alpha/beta* repeat units (Kuhn and Sene 2005) is the most abundant, representing 1.93% of the genome. The second satDNA is DBC-150 (Kuhn *et al.* 2007), with ∼110- to 150-bp-long repeat units and representing 0.8% of the genome. The third satDNA is a novel one and was named *CDSTR*138, with 138-bp-long repeat units and representing 0.23% of the genome. The fourth satDNA is *CDSTR198*, which is shared with *D. buzzatii*, but represents only 0.02% of the *D. seriema* genome.

The SSS139 satDNA, with 139-bp-long repetition units was previously described in *D. seriema* (Franco *et al.* 2008). In the *RepeatExplorer* output, we found sequences homologous to SSS139 in the 10th most abundant repeat cluster, representing 0.5% of the genome. However, detailed sequence analysis revealed that this cluster is not made of tandem repeats. Instead, most sequences correspond to an ∼30-bp SSS139 inverted fragment interrupted by a region variable both in size and identity, followed by an ∼120-bp SSS139 sequence in direct orientation. Interestingly, these variable regions or the SSS139 sequences themselves showed no similarity to any TE or satDNA family previously described. Therefore, further studies will be necessary for elucidating the nature of the SSS139 repetitive elements.

**Figure 2:**
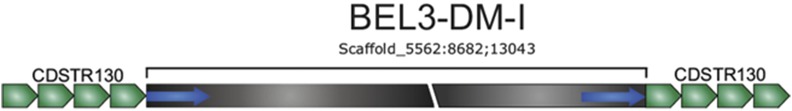
Schematic representation of the BEL3-DM-I transposable element present on RepBase, which is flanked by CDSTR130 satDNA arrays. Blue arrows represent the undescribed 185-bp-long terminal repeat of the BEL3-DM element.

We found two satDNAs in *D. mojavensis*. The most abundant is a novel one, which we named *CDSTR*130, with 130-bp-long repeat units and representing 1.63% of the genome. It is worth noting, however, that RepBase identified these sequences as a LTR BEL3_DM-I element described in *D. mojavensis* (Jurka 2012). This LTR has been characterized from *D. mojavensis* scaffold 5562 (nucleotide positions 8682–13,043 bp). However, the scrutinized analysis of 100 BEL3-DM insertions on the *D. mojavensis* genome showed that the 130-bp tandem repeats are not part of the LTR, but only flank the element in the scaffold 5562 (Figure 2). The identification of *CDSTR*130 as a satDNA highlights the importance of manual curation of the automated output provided by *RepeatExplorer*. It also explains why Melters *et al.* (2013) did not identify *CDSTR*130 as the most abundant tandem repeat family in the *D. mojavensis* genome.

The second most abundant satDNA identified in *D. mojavensis* is the *pBuM*-1 variant from the *pBuM* family (shared with *D. buzzatii* and *D. seriema*), with 185-bp-long repeats and representing 0.86% of the genome. This satDNA has been previously identified as the most abundant tandem repeat family of *D. mojavensis* by Melters *et al.* (2013).

The main features of the satDNAs identified above are summarized in Table 1 and a list containing consensus sequences from all the new satellites described in the present work can be seen in Figure S4 in File S1.

**Table 1:** Main features of satellite DNA families present on *D. buzzatii*, *D. seriema*, and *D. mojavensis* genomes

	satDNA Family	Monomer Size	GC Content (%)	Copy Number (Analyzed)	Genomic Contribution (%)	Variability (%)
*D. buzzatii*	*pBuM*	189	29	379	1.71	12.1
	*CDSTR198*	198	34	79	0.23	13.1
*D. seriema*	*pBuM-2*	370	23.9	30*^a^*	1.93	1.9*^a^*
	*DBC-150*	150	55.9	5*^b^*	0.81	11.3*^b^*
	*CDSTR138*	138	31.2	386	0.22	12.7
	*CDSTR198*	198	34.8	67	0.02	15.5
*D. mojavensis*	*CDSTR130*	130	26.2	929	1.63	13.7
	*pBuM*	185	26.5	600	0.86	4.1

a Data from Kuhn *et al.* (2008).

b Data from Kuhn *et al.* (2007).

### Cactophilic Drosophila species present the lowest satDNA content within the genus

In most analyzed *Drosophila* species, the satDNA proportion fall within the range of between 15 and 40% (Bosco *et al.* 2007; Craddock *et al.* 2016). We found that the *pBuM* and *CDSTR*130 satDNAs represent only 2.5% of the *D. mojavensis* genome. Our result, obtained from the analyses of sequence reads using *RepeatExplorer*, was very close to the 2% satDNA contribution estimated by Bosco *et al.* (2007) using flow cytometry. In addition, we also found low amounts of satDNAs in the genomes of the other two cactophilic *Drosophila*: 1.9% for *D. buzzatii* and 2.9% for *D. seriema*. The additional 1% of the *D. seriema* in relation to *D. buzzatii* is probably represented by sequences located in the microchromosome of *D. seriema*, which is larger than that of *D. buzzatii* and also contains a higher amount of satellites (pBuM-2 and DBC-150) when compared to the other chromosomes (Figure 9; Kuhn *et al.* 2007, 2009). Our data revealed that cactophilic *Drosophila* present the lowest amount of satDNAs within the *Drosophila* genus reported so far. On the other hand, the estimated contribution of repetitive DNAs (satDNA^+^TE+unclassified repeats) in the three cactophilic *Drosophila* (14–27%) is not atypical for the genus (Drosophila 12 Genomes Consortium 2007; Craddock *et al.* 2016). Future studies focusing on satDNAs of more populations and species of the *repleta* group are expected to shed light on whether the low satDNA content in cactophilic *Drosophila* is a result of selective constraints or historical events.

### Preferential satDNA repeat lengths in cactophilic Drosophila

SatDNA repeats in the three studied cactophilic *Drosophila* have lengths of 130–200 bp or between 340 and 390 bp. To confirm this result, we ran *RepeatExplorer* with sequence reads from *D. melanogaster* where satDNA repeats <10 bp are abundant. *RepeatExplorer* correctly identified them as the most abundant repetitive DNAs of *D. melanogaster* (Table S5 in File S1). Therefore, we concluded that the preferential lengths for satDNA repeats in the three cactophilic *Drosophila* are not an artifact generated by *RepeatExplorer*.

Interestingly, satDNA repeats described before the genomic era in many plant and animal species (including *Arabidopsis*, maize, humans, and many insect species) typically show basic repeat units 150–180 or 300–360 bp long (Henikoff *et al.* 2001; Heslop-Harrison *et al.* 2003). Similar repeat-length patterns have been confirmed with recent genome-wide analysis of tandem repeats in other organisms. For example, Pavlek *et al.* (2015) showed that the most abundant tandem repeat families in the beetle *Tribolium castaneum* present repeat lengths either ∼170 bp or ∼340 bp long. It is difficult to explain such preferential repeat lengths by chance. On the other hand, it is striking that these two peak units closely correspond to the length of DNA wrapped around one or two nucleosomes.

It has been hypothesized that satDNA length could play a critical role in DNA packaging by favoring nucleosome positioning (or phasing) that in turn leads to condensation of certain genomic regions, such as the heterochromatin (Fitzgerald *et al.* 1994; Henikoff *et al.* 2001). Accordingly, the preferential lengths observed in the satDNA from cactophilic *Drosophila* could be selectively constrained by a possible role in chromatin packaging.

### Satellite DNA candidates for centromeric function

The centromeres of most plant and animal species are composed of long arrays of tandemly repeated satellite DNAs (Plohl *et al.* 2014). There is increasing evidence to support a role for satDNA in centromeric function by providing motifs for centromeric-protein binding, *e.g.*, CENP-B box in alphoid human satDNA (Ohzeki *et al.* 2002), and/or by producing RNA transcripts that are necessary for centromere/kinetochore assembly (Gent and Dawe 2012; Rošić *et al.* 2014). On the other hand, centromeric satDNAs may differ greatly even between closely related species. In fact, there are several examples supporting the observation that satDNA is one of the most rapidly evolving components of the genomes. Therefore, the identification of the most likely candidate for centromere function in a species is a task that in most cases has to be performed on a case-by-case basis.

Based on data collected from several animal and plant genomes, Melters *et al.* (2013) suggested that the most abundant tandem repeat of a genome would also be the most likely candidate for centromeric location and function. To test this hypothesis, we investigated by FISH the chromosomal location of all satDNAs identified in the three cactophilic *Drosophila* sampled in the present study.

All three species share the same basic karyotype (2n = 12) consisting of four pairs of telocentric autosomes, one pair of microchromosomes, and one pair of sex chromosomes (Baimal *et al.* 1983; Kuhn *et al.* 1996; Ruiz *et al.* 1990). Heterochromatin is located in the centromeric region of all four telocentric chromosomes, along the whole microchromosomes and Y chromosome and covering approximately one third of the proximal region of the X chromosome.

**Figure 3:**
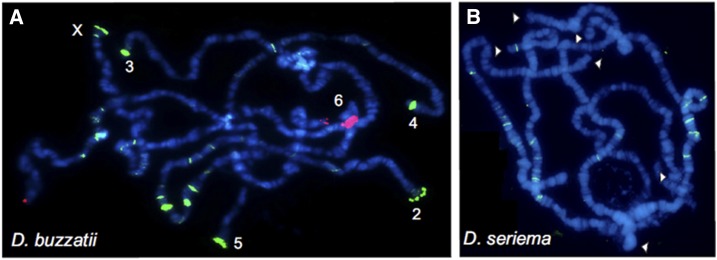
FISH on polytene chromosomes of (A) *D. buzzatii* and (B) *D. seriema* using satDNA probes for *pBuM* (red) and *CDSTR198* (green) (arrowheads indicate telomeric regions).

We identified the *pBuM*-1 alpha repeats as the most abundant satDNA of *D. buzzatii*. In a previous study, Kuhn *et al.* (2008) showed by FISH on mitotic chromosomes that *pBuM*-1 alpha repeats are located in the centromeric heterochromatin of all chromosomes except the X. In order to further investigate the chromosomal location of *pBuM*, we also hybridized a *pBuM-1* probe to the polytene chromosomes. In these chromosomes, the centromeric heterochromatin is underreplicated and forms a dense central mass in the chromocenter – a region where the centromeres of all chromosomes bundle together. We observed that the *pBuM-1* repeats are restricted to the chromocenter region (Figure 3A), therefore confirming their centromeric location. The second most abundant satDNA in *D. buzzatii* is *CDSTR198*, which was mapped by FISH in terminal and interstitial locations on metaphase chromosomes (these results are detailed below). Therefore, the most abundant satDNA of *D. buzzatii*, *i.e.*, *pBuM*, is the one showing centromeric location in most chromosomes.

**Figure 4:**
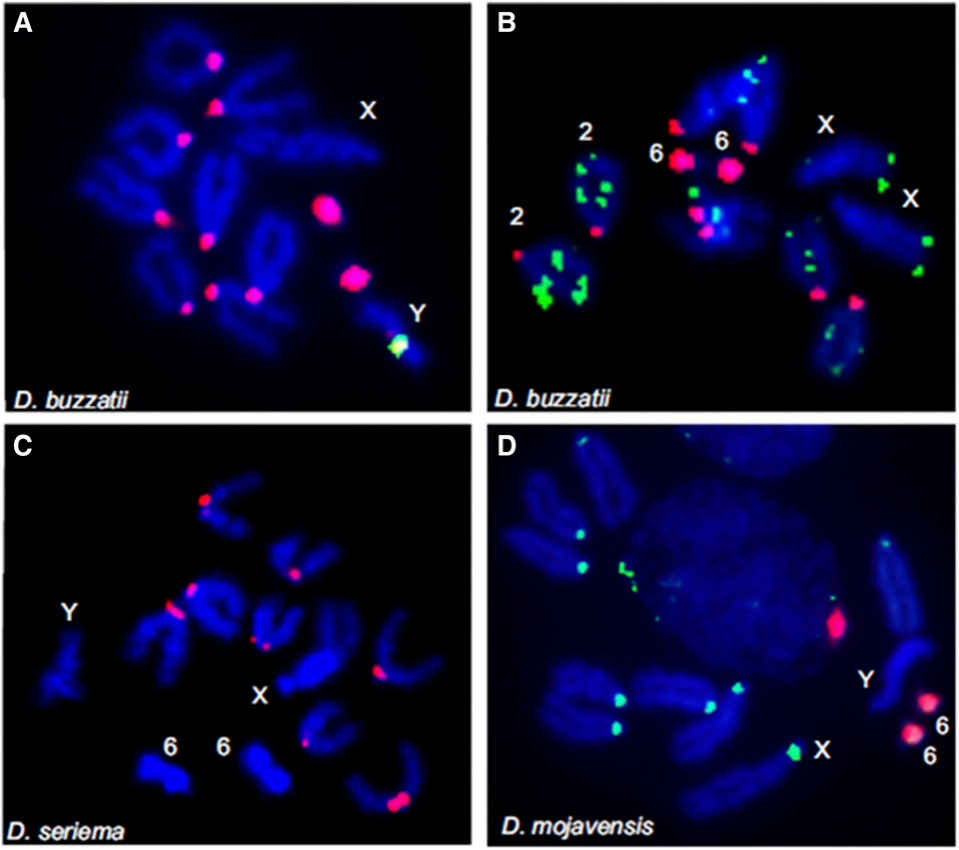
FISH on mitotic chromosomes using satellite DNA probes. (A) *pBuM-1a* (red) and pBuM-1b (green) satDNA probes on *D. buzzatii*; (B) *pBuM-1a* (red) and *CDSTR198* (green) probes on *D. buzzatii*; (C) *CDSTR138* (red) on *D. seriema*; (D) *CDSTR130* (green) and *pBuM* (red) probes on *D. mojavensis*.

In *D. seriema*, the most abundant satDNA identified was *pBuM*-2 and the second most abundant was *DBC-150*. Previous studies showed that *pBuM*-2 is located on the centromeric regions of chromosomes 2, 3, 4, and 5 and on the telomeric regions of chromosome 6 (Kuhn *et al.* 2008). *DBC-150* was found exclusively on the centromeric region of chromosome 6 (Kuhn *et al.* 2007). *CDSTR138*, the new satDNA described herein, is the third most abundant tandem repeat of this species and was mapped by FISH at the centromeric region of chromosomes 2, 3, 4, and 5 in mitotic chromosomes (Figure 4C). The centromeric location was also confirmed after FISH on polytene chromosomes, where no hybridization signals were observed outside the chromocenter (Figure 3A). The fourth identified satDNA in *D. seriema*, *CDSTR198*, showed no hybridization signal after FISH on mitotic chromosomes, confirming that it has very low copy number in this species (in contrast to *D. buzzatii*). However, we detected a few *CDSTR198* repeats in the euchromatin after FISH on polytene chromosomes (Figure 3B; see below). Therefore, all three most abundant satDNAs of *D. seriema* are part of the centromeric region of most chromosomes.

*CDSTR130* was identified as the most abundant satDNA in *D. mojavensis*; FISH on mitotic chromosomes showed that *CDSTR130* repeats are located at the centromeric region of all autosomes and the X chromosome (Figure 4D). The second most abundant satDNA is *pBuM*-1, which covered the microchromosome (chromosome 6) almost entirely (Figure 4D). Therefore, both *pBuM*-1 and *CDSTR*130 are abundant in chromosome 6. However, given the size and dot-like morphology of this chromosome in this species, it is not possible to determine which one shows centromeric location. The analysis of the polytene chromosomes showed that the two satDNAs colocalize in the chromocenter region (Figure S5 in File S1).

Based on the collection and chromosome distribution of the satDNAs discussed herein, the centromeric regions of the X chromosome of *D. buzzatii*, of the X and Y of *D. seriema*, or of the Y of *D. mojavensis* are not composed of satDNAs. Some centromeres described in plants and animals are composed of TEs (reviewed by Plohl *et al.* 2014). In *Drosophila*, DINE-1 elements (helitrons) are one of the most abundant types of TEs (Yang and Barbash 2008). Kuhn and Heslop-Harrison (2011) and Dias *et al.* (2015) showed by FISH on mitotic chromosomes that these elements are highly enriched in the sex chromosomes (including the centromeric regions) in the three analyzed species from the *repleta* and *virilis* groups. It is possible that these DINE-1 elements are the main components of the centromeres of the sex chromosomes of cactophilic *Drosophila* species.

According to *RepeatExplorer*, the genomic proportion of satDNA in *D. mojavensis* (*CDSTR130* + *pBuM*) is 2.5% (Table 1). This value is very close to the 2% satDNA contribution estimated by Bosco *et al.* (2007) using flow cytometry in the same species. According to the authors, if we split the ∼2% satDNA evenly among the *D. mojavensis* chromosomes that would result in ∼430 kb for each centromere. As noted by the authors, this value is also very close to what is considered as the minimum amount of centromeric DNA (420 kb) needed to fulfill centromeric function in *Drosophila* (Sun *et al.* 1997). In this context, Bosco *et al.* (2007) emphasized that it would be valuable to identify the centromeric satDNA of *D. mojavensis* and other *Drosophila* species to investigate whether they agree with the ∼420 kb limit observed in *D. melanogaster*.

In the present work, we found that *pBuM* and *CDSTR*130 are the main centromeric components of *D. buzzatii* and *D. mojavensis*. According to previous estimates, the male genome size of *D. buzzatii* and *D. mojavensis* is ∼170 Mb (Gregory and Johnston 2008; Romero-Soriano *et al.* 2016). Accordingly, we calculated that the bulk of centromeric satDNA in *D. buzzatii* is 2.9 Mb and in *D. mojavensis*, 2.8 Mb. If we split these values equally between the number of centromeres (= 6), each centromere will have ∼480 kb of centromeric DNA in *D. buzzatii* and ∼460 kb in *D. mojavensis*. This suggests cactophilic *Drosophila* have centromeric sizes roughly 470 kb on average, a value close to the suggested limit of 420 kb necessary for a functional centromere in *Drosophila* (Sun *et al.* 1997).

### New insights on pBuM distribution and evolution

According to previous data on the distribution of *pBuM*-1 *alpha* and *pBuM*-2 *alpha/beta* repeats in the phylogeny of *Drosophila* species from the *buzzatii* cluster (*repleta* group), it was proposed that the ancestral state of the *pBuM* satDNA family consisted of *alpha* tandem repetition units ∼190 bp long. The *alpha/beta* repeats would have been originated subsequently from an insertion of a nonhomologous sequence of 180 bp (*beta*) in an *alpha* array, resulting in a composite *alpha/beta* repeat unit that also became abundant and tandemly organized (Kuhn and Sene 2005).

We found only *alpha* repeats in the genome of *D. mojavensis*, which is consistent with the hypothesis that *alpha* repeats represent the ancestral state of the *pBuM* family. According to current estimates, the split between the *buzzatii* and *mojavensis* clusters occurred ∼11 MYA (Oliveira *et al.* 2012; Guillén *et al.* 2015), which would be the minimum age for the origin of the *pBuM* family.

In *D. seriema*, we detected only *pBuM-2* repeats, which agrees with previous DNA hybridization data (Kuhn and Sene 2005) suggesting that *pBuM-2* is the only *pBuM* subfamily present in this species. The split between *D. buzzatii* and *D. seriema* was estimated to have happened ∼3 MYA (Franco *et al.* 2010). Therefore, in the last 3 MY, it seems that there was a complete turnover from *pBuM-1* to *pBuM-2* repeats in the genome of *D. seriema*.

According to our FISH experiments on mitotic and polytene chromosomes, *pBuM* repeats are restricted to the heterochromatic regions. However, BLAST on the assembled genome (Freeze 1 Scaffolds) of *D. buzzatii* revealed fragments of *pBuM-1* repeats on three scaffolds (1, 88, and 90) that were mapped to the euchromatin from chromosomes 2, 5, and X [see Guillén *et al.* (2015) for exact location of scaffolds]. The three observed *pBuM*-1 euchromatic loci contain either a partial *pBuM-1* repeat (<189 bp) or at most two partial *pBuM-1* tandem repeats (<300 bp), and such small sizes were probably the reason they were undetected in our FISH experiments. The analysis of flanking sequences did not show evidence that these euchromatic *pBuM-1* sequences could be integral parts of TEs and the mechanism(s) responsible for their presence on euchromatin are currently unknown.

**Figure 5:**
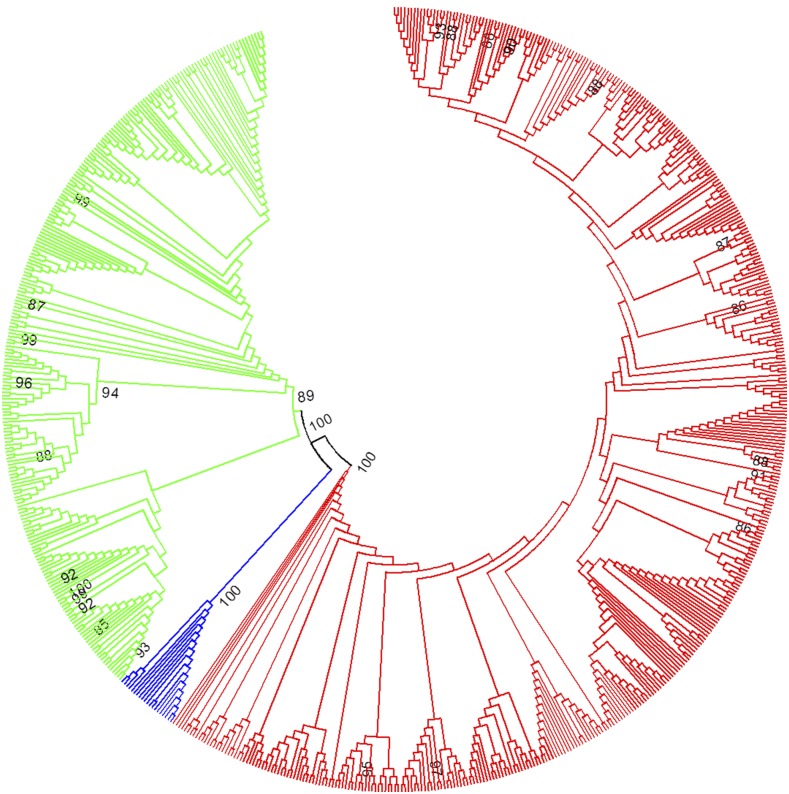
NJ tree containing a sample of *pBuM* repeats extracted from the sequenced genomes of *Drosophila buzzatii* (green), *D. seriema* (blue), and *D. mojavensis* (red). The tree was estimated using the T93 substitution model with 1000 bootstrap replicas.

Previous phylogenetic analyses of *pBuM* repeats in *D. buzzatii* and *D. seriema* showed that these repeats have been evolving according to the concerted evolution model (Kuhn and Sene 2005). In other words, repeats within each species are more similar to each other than to repeats between species. In order to test whether *pBuM* also evolved in concert in *D. mojavensis*, we constructed a NJ tree with all *pBuM* repeats extracted from *D. buzzatii*, *D. seriema*, and *D. mojavensis* (Figure 5). The NJ tree revealed *pBuM* repeats from each species allocated in species-specific branches, indicating that *pBuM* has been evolving in a concerted manner in the last 11 MY.

### The presence of pBuM in the nonrecombining Y allowed independent homogenization

In a previous report, the analysis of 63 *pBuM*-1 *alpha* repeats from *D. buzzatii* revealed very low levels of interrepeat variability (4.2% on average), indicating that, despite multiple chromosomal location, *pBuM* arrays have been efficiently homogenized at the intraspecific level (Kuhn *et al.* 1999). However, one repeat (Juan/4) showed atypical levels of nucleotide divergence in comparison to the remaining repeats (22% on average). Kuhn *et al.* (1999) suggested that this repeat may belong to another, less abundant, *pBuM* subfamily.

**Figure 6:**
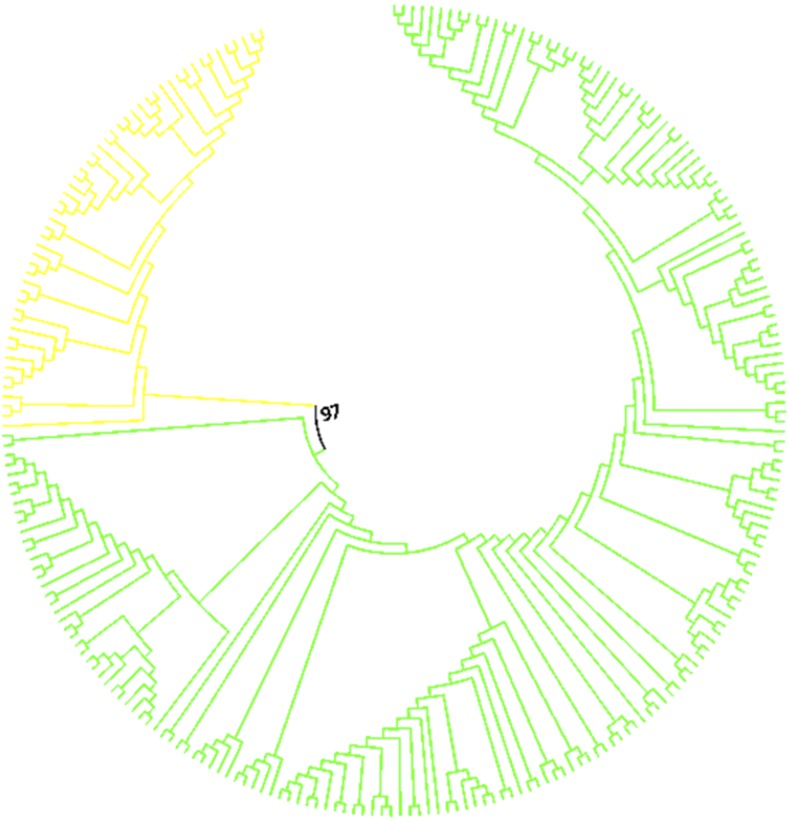
NJ tree of *pBuM* satDNA repeats retrieved from the *D. buzzatii* assembled genome and previously described in Kuhn *et al.* (1999). Colored branches evidence Y chromosome-specific arrays (yellow) when compared to autosomal arrays (green). The tree was estimated using the T93 substitution model with 1000 bootstrap replicas.

In the present work, we retrieved a sample of 247 *pBuM-1* repeats from the sequenced genome of *D. buzzatii* and used them to construct a NJ tree. The resulting tree split the repeats into two main branches (Figure 6). The major one, containing 194 repeats, contains the “typical” *pBuM-1* repeats, described in Kuhn *et al.* (1999). The second minor branch, with 53 repeats, contains “Juan/4-like” *pBuM-1* repeats. Between the two groups, the nucleotide difference is 24.2%.

These data are consistent with the hypothesis of two *pBuM* subfamilies being present in the *D. buzzatii* genome. Herein, we will name them as *pBuM*-1a (typical) and *pBuM*-1b (“Juan/4-like”). All the data generated so far about *pBuM* from *D. buzzatii* (including chromosomal location) concern the typical *pBuM*-1a repeat variant. There are several diagnostic nucleotide substitutions that allow discrimination between *pBuM* repeats from these two subfamilies. Such a situation allowed us to design oligonucleotides to specifically amplify *pBuM*-1b repeats by PCR for probe preparation. We then performed double-FISH with *pBuM*-1a and pBuM-1b on *D. buzzatii* mitotic chromosomes. The *pBuM*-1a probe showed the same multichromosomal distribution as described before. However, the *pBuM*-1b probe hybridized specifically to the Y chromosome (Figure 4A).

According to the model of concerted evolution, intraspecific homogenization of repeats occurs by recombination events such as unequal crossing over and gene conversion (Dover 1982; Dover and Tautz 1986). There is also some evidence suggesting that different arrays on the same or in different chromosomes may experience independent homogenization for arrays- or chromosomal-specific repeat variants (*i.e.*, intragenomic concerted evolution) (Kuhn *et al.* 2012; Larracuente 2014; Khost *et al.* 2017). In this context, it is expected that arrays with tandem repeats on nonrecombining chromosomes, such as the Y, would be specially subjected to independent homogenization. This is most likely the reason for the existence of a different *pBuM* subfamily (*pBuM*-1b) on the Y chromosome of *D. buzzatii*. Furthermore, empirical and experimental data showed that low recombination is expected to increase interrepeat variability (Stephan and Cho 1994; Navajas-Pérez *et al.* 2006; Kuhn *et al.* 2007). In fact, *pBuM*-1a repeats had a nucleotide difference of 12%, while the *pBuM*-1b repeats (restricted to the Y chromosome) showed a higher variability of 17%.

### The CDSTR198 satDNA shows terminal and dispersed distribution

The *CDSTR198* satDNA was found in *D. buzzatii* and *D. seriema*, but with marked quantitative differences (0.23% in *D. buzzatii* and 0.02% in *D. seriema*). FISH on *D. buzzatii* mitotic chromosomes revealed that this satDNA is located in the terminal regions of chromosomes 2, 3, 4, 5, and X but also spread along euchromatic regions (Figure 4A). FISH on polytene chromosomes of the same species revealed strong hybridization signals in the telomeric regions of chromosomes 2, 5, and X, and in subtelomeric regions of chromosomes 3 and 4 (Figure 3A). Moreover, we detected the presence of *CDSTR198* repeats along euchromatic regions of all chromosomes, except on the microchromosome. We found the highest number of *CDSTR198* euchromatic signals concentrated in chromosomes 2 and 5 (Figure 3A). Similar results were also obtained by an overall analysis of 37 *CDSTR198* euchromatic arrays present in the *D. buzzatti* assembled genome (Table S6 in File S1). Interestingly, this analysis showed an equal number of euchromatic arrays present on chromosomes 2 and 3 (11 arrays each), followed by chromosomes 4 and 5 (six arrays each). The fewer euchromatic arrays found in the *D. buzzatii* genome may result from the computational challenge of repetitive element assembly (Treangen and Salzberg 2012), reinforcing the need for hybridization experiments of satDNA families spread throughout euchromatin. In line with this, it is relevant to suggest that some *CDSTR198* arrays identified by FISH may be absent on assembled genomes. FISH on polytene chromosomes of *D. seriema* showed *CDSTR198* located only in a few euchromatic sites (Figure 3B).

In contrast to TEs, satDNAs do not have the ability to transpose by themselves. However, there are some reported examples showing that TEs may act as a substrate for satDNA emergence and mobility (Dias *et al.* 2015; Meštrović *et al.* 2015; Satović *et al.* 2016). We created a database containing the 500-bp sequences immediately before and after each *CDSTR198* array (37 in total; Table S6 in File S1) found in the assembled scaffolds of *D. buzzatii*. Comparative analysis of all flanking sequences did not show association to a specific TE or TE family or to any other specific sequence common to all arrays. These results raise the question about the dispersion mechanism of *CDSTR198* in the *D. buzzati* genome.

Tandemly repeated sequences may undergo small recombination events involving copies of the same array in the same orientation. These events may result in the formation of extrachromosomal circular DNAs (*eccDNAs*) (Cohen and Segal 2009). The occasional presence of a replication-initiating region may provide further amplification and new *eccDNA* copies. Apparently, these *eccDNAs* can be inserted again into the genome by recombination. This mechanism was proposed to explain the dispersion of copies of the *satDNA* TCAST2 in *Tribolium castaneum* (Brajković *et al.* 2012), as well as of the *D. melanogaster 1.688* satDNA (Cohen and Segal 2009), which also show an euchromatic dispersed distribution (Kuhn *et al.* 2012). In order to test this hypothesis, it would be interesting to look for the presence of *eccDNA*-containing *CDSTR198* repeats in *D. buzzatii*.

### CDSTR198 satDNA may contribute to telomeric function in D. buzzatii

Unlike most eukaryotes, *Drosophila* telomeric regions are maintained by a sequence complex organized in three subdomains: (i) arrays of TEs (Het-A/TART) responsible for maintaining telomeric sequences; (ii) telomere-associated sequences (TAS), formed by complex repetitive sequences, usually satDNAs, and (iii) a protein complex HOAP required for telomere stability (Silva-Sousa and Casacuberta 2012). Although the structure of telomeres is conserved among all *Drosophila* species, the TEs and TAS sequences are highly variable even among phylogenetically close species (Villasante *et al.* 2007). Based on the widespread presence of TAS in *Drosophila* and other species (including humans), Biessmann *et al.* (2000) proposed that homologous recombination between terminal satDNA repeats could have been an “ancient” mechanism for telomere extension. Today, TAS regions probably function as a buffer zone between the telomeres and internal chromosome domains (Sharma and Raina 2005).

We could not identify conserved domains for telomeric Het-A and TART TEs in the sequenced genome of *D. buzzatii*, even though these TEs were described in *D. mojavensis* and *D. virilis* (Villasante *et al.* 2007). Similarly, a recent screening of the *D. buzzatii* sequenced genome for the whole TE content did not identify Het-A or TART elements (Rius *et al.* 2016). The apparent absence of Het-A and TART in *D. buzzatii* may be related to the high evolutionary rate of these sequences (Villasante *et al.* 2007). Alternatively, there may be a different mechanism for telomere elongation operating in this species.

The *CDSTR198* satDNA is located in the telomeric and subtelomeric regions of five (out of six) chromosomes of *D. buzzatii* (Figure 3A and Figure 4B). The presence of *CDSTR198* in the telomeres associated with the apparent absence of Het-A and TART sequences open the possibility that *CDSTR198* plays a role in telomere elongation through a recombination-based mechanism (*e.g.*, unequal crossing over). Although not described in *Drosophila*, tandem repeat sequences are responsible for maintaining telomeres in the dipterous genus *Chironomus* (Löpez *et al.* 1996).

It is important to mention that a similar scenario described herein for the *CDSTR198* of *D. buzzatii* was previously reported for *D. virilis*, which belongs to the *virilis* group. In this noncactophilic species, the terminal location of the *pvB370 satDNA* associated with the absence of telomere transposons led Biessmann *et al.* (2000) to propose the involvement of this satDNA in telomere elongation. However, TART-like and HeT-like elements were later described in the terminal regions of *D. virilis*, opening the possibility that these elements also participate in telomeric elongation in this species (Casacuberta and Pardue 2003; Pardue *et al.* 2005).

### pBuM and CDSTR130 show regions of interspersed distribution in the microchromosomes

**Figure 7 (A and B):**
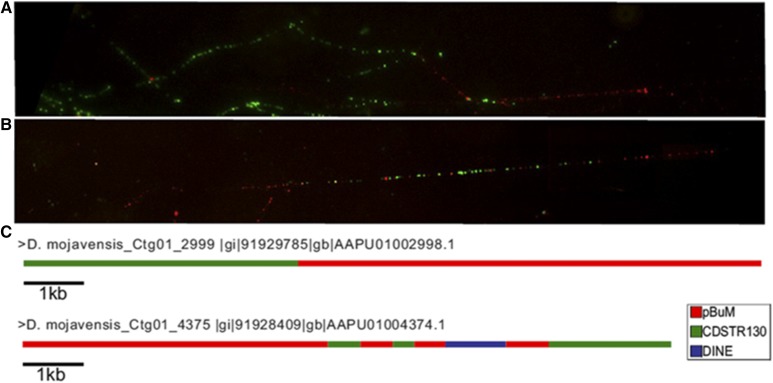
FISH with *CDSTR*130 (green) and pBuM (red) probes onto extended DNA fibers of *D. mojavensis*. (C) Schematic representation of *CDSTR130* and *pBuM* organization found on contigs *Ctg01_2999*(AAPU01002998.1) and *Ctg01_4375*(AAPU01004374.1) retrieved from the *D. mojavensis* assembled genome.

FISH with *CDSTR*130 and *pBuM* probes on *D. mojavensis* mitotic chromosomes revealed that these two satDNA colocalize on the microchromosome. In order to further investigate how these two satDNAs are organized, we performed double-FISH experiments on extended DNA fibers. We observed strong hybridization signals in fibers showing *CDSTR130* long arrays followed by *pBuM* long arrays (Figure 7A). However, in some DNA fibers hybridization signals indicated an interspersed organization of both satDNAs (Figure 7B). These results were also confirmed in the analysis of *D. mojavensis* assembled contigs (Figure 7C). For example, the contig 2999 (AAPU01002998.1) is composed of 4435 bp of *CDSTR130* copies adjacent to a *pBuM* array of 7716 bp. In the contig 4375 (AAPU01004374.1), we observed different arrays of *pBuM* and *CDSTR130* interspersed with each other (Figure 7C).

Nonhomologous satDNAs located in the same chromosome region are usually organized in separate arrays (*e.g.*, Shiels *et al.* 1997; Lohe *et al.* 1993; Sun *et al.* 2003). However, there are some reports showing interspersion of repeats from different satellites (*e.g.*, Žinić *et al.* 2000; Alkhimova *et al.* 2004; Wei *et al.* 2014). It has been suggested that interspersion between repeats may give rise to new higher order repeat structures (Mravinac and Plohl 2007; Wei *et al.* 2014). In a previous study conducted in cactophilic *Drosophila* species, Kuhn *et al.* (2009) showed high levels of interspersion between *pBuM* and DBC-150 in at least two species of the *buzzatii* cluster (*D. gouveai* and *D. antonietae*). Interestingly, such pattern was also observed in the microchromosomes. According to Kuhn *et al.* (2009), interspersion of repeats from nonhomologous satellites in the microchromosomes could be related to the peculiar characteristics of these chromosomes, such as highly heterochromatic nature and low content of genes, which could allow a more flexible interplay between repetitive elements without deleterious effects.

### Differential transcription of cactophilic Drosophila satDNAs

SatDNAs do not code for proteins and have been traditionally viewed as “junk DNAs.” However, there is a growing number of studies showing satDNA transcription activity from yeast to mammals, and the biological function of these transcripts has now started to be appreciated. For example, satDNA transcripts were shown to be involved in heterochromatin assembly, kinetochore formation, and gene regulation (reviewed by Biscotti *et al.* 2015; Ferreira *et al.* 2015). Moreover, transcription of satDNAs is usually gender or stage specific and is often associated with differentiation and development (Usakin *et al.* 2007; Pecinka *et al.* 2010).

**Figure 8:**
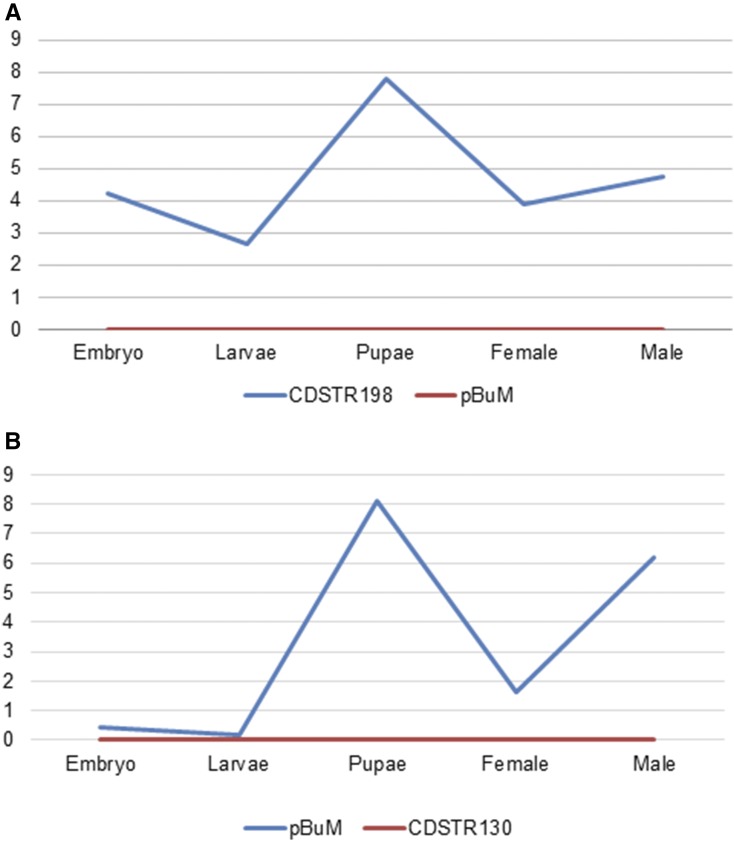
Transcription profile of satDNA families in *D. buzzatii* (A) and *D. mojavensis* (B) at five different developmental stages. Counts were normalized to one million reads.

Herein, we investigated whether the satDNAs that we analyzed are transcribed by mapping the satDNA consensus sequences on the available RNA-seq data from *D. buzzatii* and *D. mojavensis* (Guillén *et al.* 2015; Rius *et al.* 2016). Read counts were calculated for embryos, third-staged larvae, pupae, and for male and female adult carcasses (Figure 8) (see *Materials and Methods*).

Our analysis did not identify transcripts from the most abundant satDNAs in the genome of *D. buzzatii* and *D. mojavensis*, *pBuM* and *CDSTR*130, respectively. As discussed previously, both are the main candidates for centromeric function in these species. This result was unexpected because previous studies in *D. melanogaster* showed that centromeric satellite RNAs in the form of long polyadenylated products play an important role in the formation of the kinetochore (Topp *et al.* 2004; Chan *et al.* 2012; Rošić *et al.* 2014). However, our results do not exclude the possibility that *pBuM* and *CDSTR*130 are transcribed. In this case, the absence of satDNA transcripts may be related to the methodology used for RNA extraction that preferentially captures poly(A) sequences. For example, satDNA transcripts of *D. melanogaster* involve non-coding RNAs that do not have poly(A) tails (Usakin *et al.* 2007).

**Figure 9:**
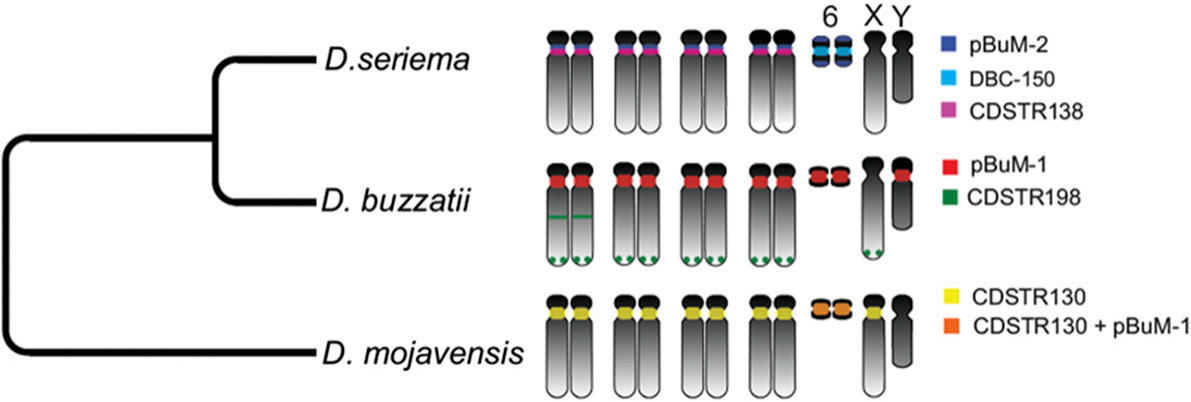
Representative ideogram showing the chromosomal localization of all satDNAs identified in *D. buzzatii*, *D. seriema*, and *D. mojavensis*.

Conversely, in all five analyzed tissues we detected transcripts derived from the *CDSTR198* satDNA of *D. buzzatii* and from the *pBuM* satDNA of *D. mojavensis*. In both cases, the transcripts were particularly abundant in tissues from pupae and males. Interestingly, these two satDNAs are located in different genomic environments: while *CDSTR198* arrays are located at several euchromatic loci (including some close to genes; Table S7 in File S1) in several *D. buzzatii* chromosomes, pBuM is exclusively located in the heterochromatic microchromosome of *D. mojavensis* (Figure 9). Future studies will be needed to address whether these transcripts participate in chromatin modulation and/or if they affect the transcription of neighboring genes, as observed for satDNA transcripts of *Drosophila* and other organisms (Menon *et al.* 2014; Feliciello *et al.* 2015).

## Supplementary Material

Supplemental material is available online at www.g3journal.org/lookup/suppl/doi:10.1534/g3.117.042093/-/DC1.
