# Supplementary material for: Dissecting the Satellite DNA Landscape in Three Cactophilic Drosophila Sequenced Genomes
Source: G3 (Bethesda). 2017 Jun 28;7(8):2831–43. doi: 10.1534/g3.117.042093 (PMC5555486; doi:10.1534/g3.117.042093)
Supplement: Supplementary file 1 [file 2831File002.pdf]

Supplementary Material

Supplementary Figures Legends:

Fig. S1. Repetitive clusters (n=122) in *D. buzzatii* identified by RepeatExplorer after clusterization of 270366 reads. Together, these clusters represent 14.7% of the genome (identified by the yellow traced line). Each bar in the graphic represents a cluster of similar reads. The pBuM-1 and CDSTR198 satellite DNAs are indicated.

Fig. S2. Repetitive clusters (n=328) in *D. seriema* identified by RepeatExplorer after clusterization of 526010 reads. Together, these clusters represent 26.9% of the genome (identified by the yellow traced line). Each bar in the graphic represents a cluster of similar reads. The pBuM-2, DBC-150 and *CDSTR138* satellite DNAs are indicated.

Fig. S3. Repetitive clusters (n=217) in *D. mojavensis* identified by RepeatExplorer after clusterization of 323342 reads. Together, these clusters represent 14.9% of the genome (identified by the yellow traced line). Each bar in the graphic represents a cluster of similar reads. The *CDSTR130* and pBuM-1 satellite DNAs are indicated.

Fig. S4. SatDNA consensus sequences from *D. buzzatii*, *D. seriema* and *D. mojavensis*.

Fig. S5. FISH on polytene chromosomes: **(A)** *CDSTR130* (green) and *pBuM* (red) satDNAs probes on *D. mojavensis*, and **(B)** *CDSTR138* satDNA probe (red) on *D. seriema*.

Supplementary Tables Legends:

Table S1. List of primers used in the present study.

Table S2. Description of all clusters retrieved from 1834708 reads of *D. buzzatii* by RepeatExplorer. The satDNA families analyzed in this study are highlighted in bold red.

Table S3. Description of all clusters retrieved from 2144275 reads of *D. seriema* by RepeatExplorer. The satDNA families analyzed in this study are highlighted in bold red.

Table S4. Description of all clusters retrieved from 2174346 reads of *D. mojavensis* by RepeatExplorer. The satDNA families analyzed in this study are highlighted in bold red.

Table S5. Description of the ten most abundant clusters of the *D. melanogaster* genome identified by RepeatExplorer. The satDNA families with monomer lengths smaller than 50 bp are highlighted in bold.

Table S6. Main features of 37 *CDSTR198* arrays located on euchromatic regions and their chromosome location according to GenomeBrowser analysis.

Table S7. List of genes associated with *CDSTR198* arrays and their relative positions in relation to *CDSTR198*.

Supplementary Figure 1.

D. buzzatii

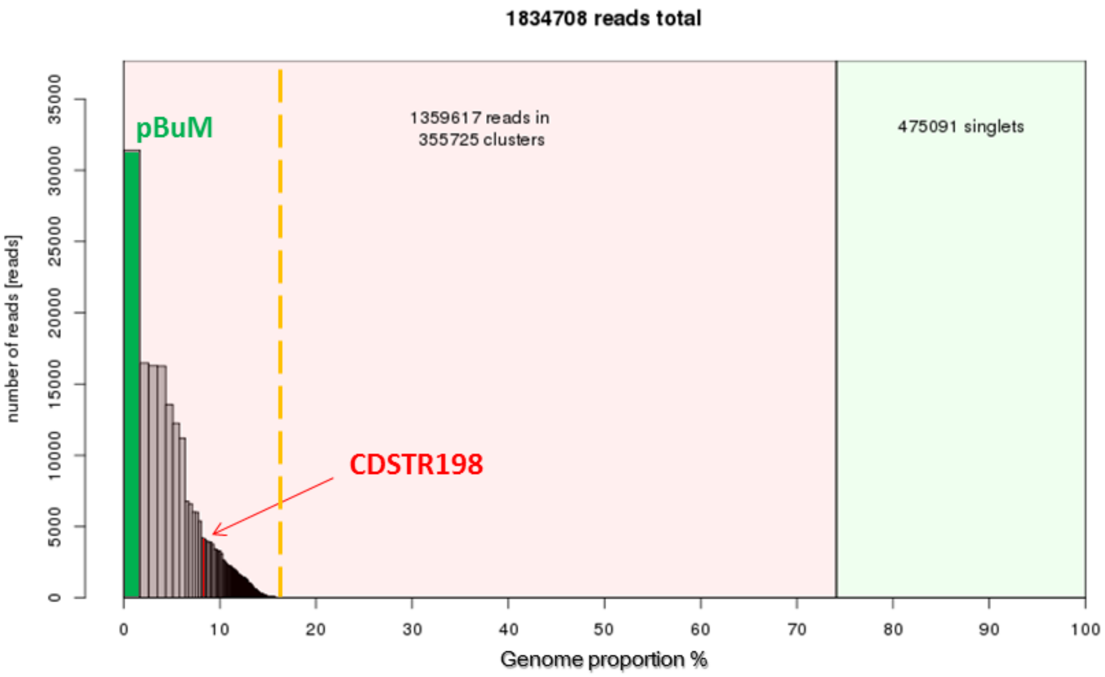

Fig. S1. Repetitive clusters (n=122) in *D. buzzatii* identified by RepeatExplorer after clusterization of 270366 reads. Together, these clusters represent 14.7% of the genome (identified by the yellow traced line). Each bar in the graphic represents a cluster of similar reads. The pBuM-1 and CDSTR198 satellite DNAs are indicated.

Supplementary Figure 2.

D. seriema

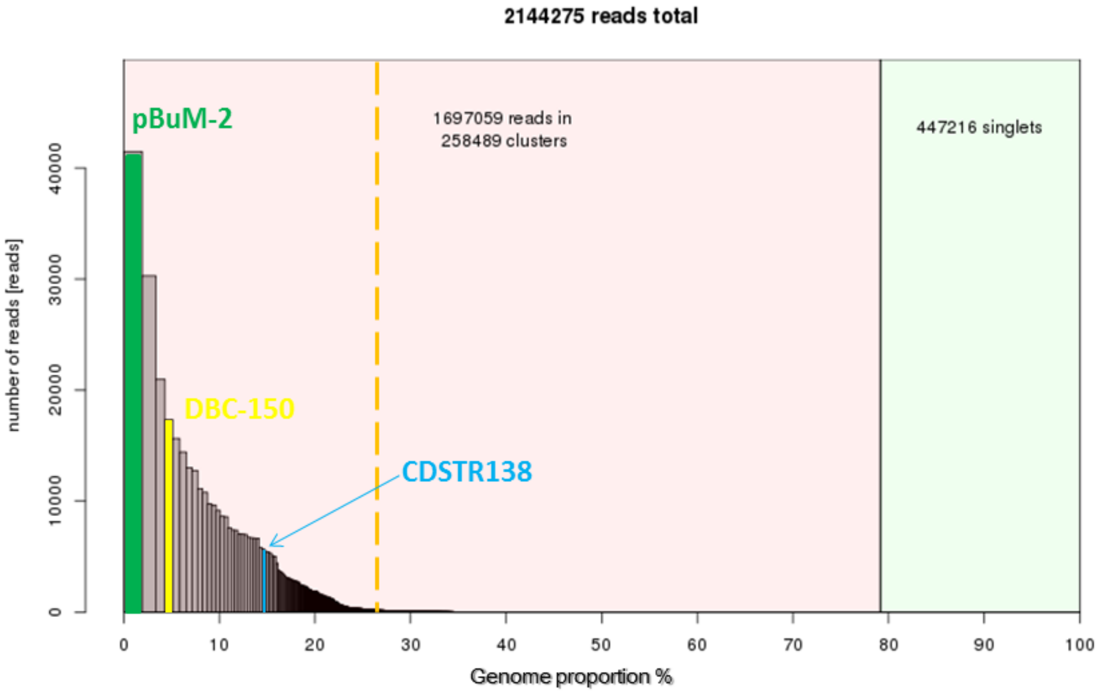

Fig. S2. Repetitive clusters (n=328) in *D. seriema* identified by RepeatExplorer after clusterization of 526010 reads. Together, these clusters represent 26.9% of the genome (identified by the yellow traced line). Each bar in the graphic represents a cluster of similar reads. The pBuM-2, DBC-150 and *CDSTR138* satellite DNAs are indicated.

Supplementary Figure 3

D. mojavensis

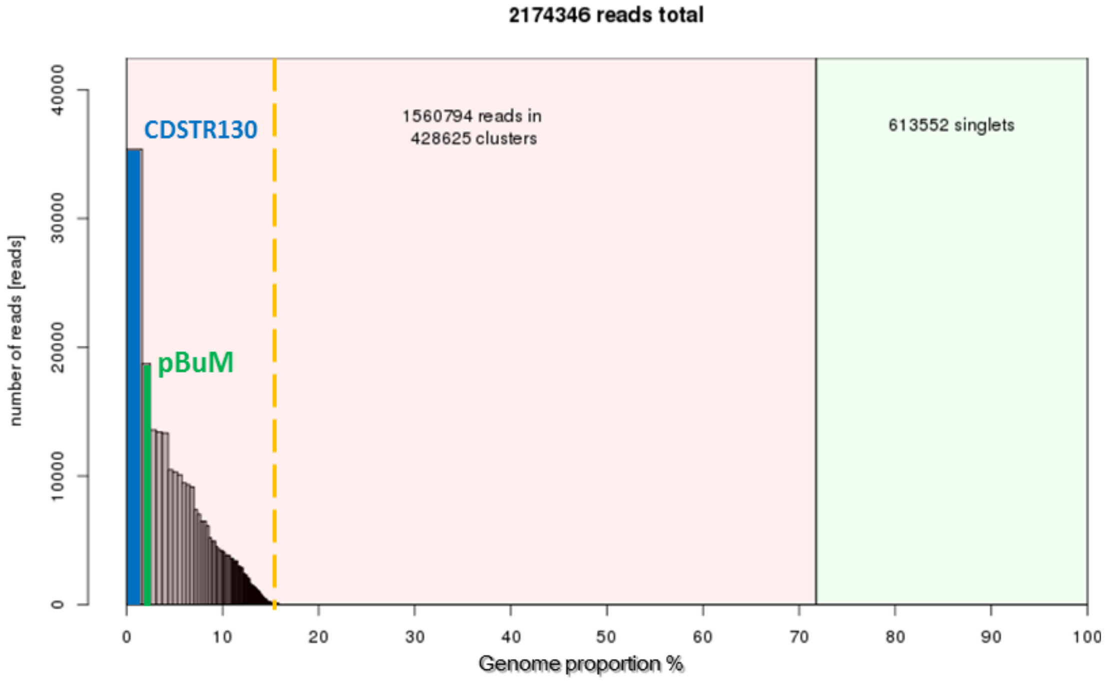

Fig. S3. Repetitive clusters (n=217) in *D. mojavensis* identified by RepeatExplorer after clusterization of 323342 reads. Together, these clusters represent 14.9% of the genome (identified by the yellow traced line). Each bar in the graphic represents a cluster of similar reads. The *CDSTR130* and pBuM-1 satellite DNAs are indicated.

Supplementary Figure 4

```
>CDSTR130_SatDNA_D.mojavensis_MF170235
CAATAGAGAAAAATTGCAAAATGTGATGGAAAATCAAAAAAAGCAAAGGGATTTTATATTTTAAAG
CTTTGATAACAAAATATTGAAAGATATTCGGCAATAAACTGGTAAATATTG
>CDSTR138_SatDNA_D.seriema_MF170236
CAACTGTTGATTTTTGTATATAGAATACGAATAAAATCAAATATATGGTGTAGAAGAGTATATTTG
CGCCGATTTACCATGAACATAACACTAGTTTGATTTTTTATTCATTTATAGTGAGTATAGGCATAT
CTGGGC
>CDSTR198_SatDNA_D.buzzatii_MF170237
AAGGTAGAAAGGTAGTTGGTGAGATAAACCAGAAAAAGAGCTAAAAACGGCTAAAAACGGCTAGAA
AATAGCCAGAAAGGTAGATTGAACATTAATGGGCAAATGGATGGATAAATAAGACTGGTCATCATC
CAATGAACAGAATCATGATTAAGAGATAGAAATATGATTAGAAAGTAGGATAGAAAGGTTAGAAAG
>pBuM-1a_SatDNA_D.buzzatii_MF170238
GCAAAAGACTCCGTCAATTAGAAAACAAAAAATGTTATAGTTTTGAGGATTAACCGGCAAAAACCG
TATTATTTGTCATTTGATTTCTTTATGGAATACCGTTTTAGAAGCGTCTTTTATCGTATTACTCAG
ATATATCTTAAGATTTAGCATAATCTAAGAACTTTTTGAAATATTCACATTTGTCCA
>pBuM-1b_SatDNA_D.buzzatii_Y_MF170239
TAAAATTACTACTTGAAACTAGAAAGAAAGAAAAGTTATAGTTTTGAGGTTTAACCGGCAAAAATC
GTATTATTTATCATTAGATTTCTTTATAGCATGCCGTTTATAAGCGTGTCTTATCGGATTATTCAG
ATATATTGCAAAATTTAACATAGCTCGAGACCTTTTTGAAATATTAACATTAATCCA
>pBuM-1_SatDNA_D.mojavensis_MF170240
CCTGAAATGCGGTATAAGATCAGAAAATTGACTTTTTTCTATGTATAACAGGCAATAACAGGACTA
TTTCGGGGCCGATTTCAGTACGACTTTCTTTGTTGGAAGCATCTTTTAGAGCCCTATCTATTGACG
TATTCAGATTTTCAATGGGCCAACAACTTTTGAAATATTTACATTATTATCCC
```

Fig. S4. satDNA consensus sequences from *D. buzzatii, D. seriema* and *D. mojavensis*.

Supplementary Figure 5.

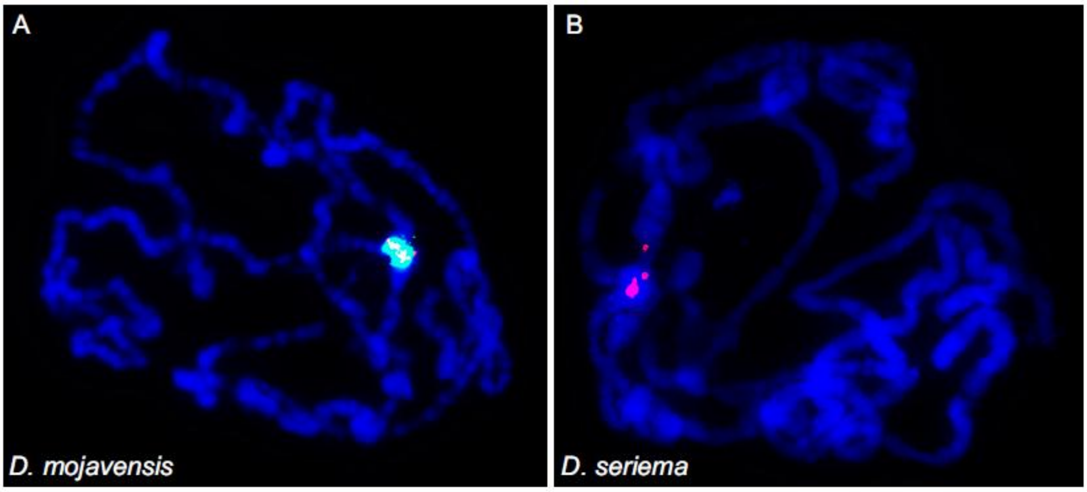

Fig. S5. FISH on polytene chromosomes: **(A)** *CDSTR130* (green) and *pBuM* (red) satDNAs probes on *D. mojavensis*, and **(B)** *CDSTR138* satDNA probe (red) on *D. seriema*.

Supplementary Table 1. List of primers used in the present study.

	Primer sets	Primer sequences
D. buzzatii	*pBuM-a*	Fwd: CAATTAGAAAACAGGAAATTG<br>Rev: GACGGAGTCTTTTGCTGGACAA
	pBuM-1b	Fwd: GAAACTAGAAGGAAATAAACG<br>Rev: CAAGGAGTCCTTTTATGGATTA
	CDSTR198	Fwd: GGTAGATTGAACACTAATGGGC<br>Rev: CCTTTCTGCCTTTTTTCTAGCCG
D. seriema	*CDSTR138*	Fwd:CCGATTTTCCATGAACATAACC<br>Rev: CGCAAATATACTCTACTACACC
D. mojavensis	*CDSTR130*	Fwd: CAATAGAGGCAAATGAATTTAG<br>Rev: CATTACTAACCAGTTTATTGCCG
	pBuM-1a	Fwd: GCCGATTTCAGTACGACTTACTTCG<br>Rev: CCAGAAATAGTCCTGTTATTGCCTG

Table S2. Description of all clusters retrieved from 1834708 reads of *D. buzzatii* by RepeatExplorer. The satDNA families analyzed in this study are highlighted in bold red.

Cluster	Genome Proportion %	RepeatMasker Annotation	Reads number on each cluster	Outside reads with similarity %
**CL1**	**1.710**	***pBuM-1***	**31409**	**0.00038**
**CL2**	0.898	RC.Helitron	16475	0.19870
**CL3**	0.888	DNA.hAT.Pegasus	16285	2.70400
**CL4**	0.886	DNA.hAT.hobo	16263	3.69400
**CL5**	0.739	Helitron	13567	4.80500
**CL6**	0.668	RC.Helitron	12252	8.96000
**CL7**	0.611	Non-LTR CR1	11208	0.11510
**CL8**	0.370	Galileo	6790	0.55450
**CL9**	0.360	Homo6	6601	6.44000
**CL10**	0.328	LINE-like	6024	0.03731
**CL11**	0.327	Galileo	6007	0.49990
**CL12**	0.294	rRNA	5393	0.05911
**CL13**	**0.229**	***CDSTR198***	**4202**	**0.00650**
**CL14**	0.226	Unclassified	4142	0.08527
**CL15**	0.217	LTR BEL	3982	0.06588
**CL16**	0.215	PERI	3940	2.02800
**CL17**	0.213	LTR BEL	3914	0.30090
**CL18**	0.205	Galileo/Helitron	3766	6.62800
**CL19**	0.188	rRNA	3442	0.16500
**CL20**	0.185	LTRGypsy	3387	0.08860
**CL21**	0.181	Non-LTR_R1	3317	0.05204
**CL22**	0.179	LTR Gypsy/Minicopia	3290	0.05014
**CL23**	0.170	LTR Gypsy	3122	0.03179
**CL24**	0.145	LTR BEL	2653	0.04116
**CL25**	0.143	LTR Gypsy	2622	0.42640
**CL26**	0.136	LTR Osvaldo	2493	0.12570
**CL27**	0.129	PARISa	2364	0.08243
**CL28**	0.125	INVADER6_I	2292	0.14160
**CL29**	0.124	LTR BEL	2279	0.42260
**CL30**	0.121	Galileo	2214	0.29560
**CL31**	0.120	LTR Copia	2198	0.28850
**CL32**	0.113	LTR Gypsy	2070	0.13140
**CL33**	0.110	RC.Helitron	2025	0.28540
**CL34**	0.109	LTR Osvaldo	1996	0.06629
**CL35**	0.104	Unclassified	1917	0.27710
**CL36**	0.100	Unclassified	1835	0.05137
**CL37**	0.095	Non-LTR Jockey	1736	0.02687
**CL38**	0.089	LTR BEL	1640	0.57820
**CL39**	0.089	Non-LTR R1	1632	0.21130
**CL40**	0.087	LTRBEL	1592	0.02742
**CL41**	0.084	Homo9	1537	0.29000
**CL42**	0.082	LTR Gypsy	1501	0.03540

**CL43**	0.079	Non-LTR Jockey	1451	0.06051
**CL44**	0.078	LTR Copia	1437	0.00347
**CL45**	0.078	Non-LTR Jockey	1428	0.02450
**CL46**	0.077	LTR Copia	1411	0.11840
**CL47**	0.074	Non-LTR_I	1351	0.07546
**CL48**	0.073	DNA.TcMar.Tc1	1340	0.52960
**CL49**	0.069	Non-LTR Jockey	1260	0.38600
**CL50**	0.061	Unclassified	1126	0.12680
**CL51**	0.059	Non-LTR Jockey	1076	0.23030
**CL52**	0.058	LTR BEL	1066	0.03978
**CL53**	0.056	LTR Copia	1019	0.15520
**CL54**	0.055	DNA.CMC.Transib	1015	0.49800
**CL55**	0.054	LTR Gypsy	987	0.00000
**CL56**	0.053	Non-LTR RTE.BovB	982	0.00000
**CL57**	0.050	LTR Gypsy	909	0.12640
**CL58**	0.049	DNA.TcMar.Tc1	891	0.20170
**CL59**	0.045	Non-LTR Jockey	828	0.03882
**CL60**	0.043	DNA.hAT-9	795	0.06751
**CL61**	0.038	Unclassified	705	0.68000
**CL62**	0.038	RNA	699	0.00454
**CL63**	0.037	RC.Helitron	687	0.09555
**CL64**	0.035	LTR Copia	644	0.00000
**CL65**	0.035	DNA.hAT.hobo	639	0.30280
**CL66**	0.035	LTR Copia	636	0.07575
**CL67**	0.034	LTR Gypsy	627	0.10790
**CL68**	0.034	Unclassified	625	0.19720
**CL69**	0.033	Galileo	604	0.25630
**CL70**	0.030	DNA.TcMar.Tc1	560	0.07143
**CL71**	0.030	RC.Helitron	543	0.03627
**CL72**	0.029	Unclassified	530	0.00000
**CL73**	0.028	DNA.TcMar.Tc1	509	0.22400
**CL74**	0.026	Non-LTR Jockey	480	0.47450
**CL75**	0.026	Unclassified	477	0.19390
**CL76**	0.025	LTR Gypsy	450	0.00000
**CL77**	0.023	LTR Copia	430	0.00000
**CL78**	0.023	LTR Gypsy	429	0.36040
**CL79**	0.022	DNA.hAT.Pegasus	412	0.08403
**CL80**	0.022	Rep2-DF	396	0.13160
**CL81**	0.021	LINE jockey	383	0.00000
**CL82**	0.021	Unclassified	382	0.00000
**CL83**	0.021	DNA.TcMar.Tc1	376	0.27770
**CL84**	0.020	DNA.hAT.Ac	375	0.05963
**CL85**	0.020	LTR Copia	368	0.04246
**CL86**	0.019	LTR Gypsy	344	0.28300
**CL87**	0.018	DNA.TcMar.Tc1	335	0.38710
**CL88**	0.018	LTRGypsy	333	0.05133
**CL89**	0.018	LTR Copia	332	0.07948
**CL90**	0.017	LTR Gypsy	314	0.33650

**CL91**	0.017	LTR Gypsy	314	1.91400
**CL92**	0.017	DNA.PiggyBac	312	0.00000
**CL93**	0.017	LTR BEL	309	0.00000
**CL94**	0.017	DNA.hAT.hobo	309	0.05537
**CL95**	0.017	LTR Copia	307	0.20480
**CL96**	0.016	Non-LTR Jockey	299	0.32750
**CL97**	0.016	LTR Gypsy	298	0.15710
**CL98**	0.016	DNA.CMC.Transib	297	1.41100
**CL99**	0.016	Non-LTR Jockey	293	0.00000
**CL100**	0.016	DNA.TcMar.Tc1	291	0.47050
**CL101**	0.016	LTR Gypsy	289	0.00000
**CL102**	0.015	Unclassified	268	0.00000
**CL103**	0.015	Unclassified	267	0.25610
**CL104**	0.014	Unclassified	259	0.00000
**CL105**	0.014	LTR Gypsy	249	0.07072
**CL106**	0.013	LTR Gypsy	245	0.09872
**CL107**	0.013	Unclassified	244	0.23550
**CL108**	0.013	Unclassified	241	0.11280
**CL109**	0.013	LTR Gypsy	235	0.41250
**CL110**	0.013	LTR BEL	231	0.00000
**CL111**	0.012	LTR Gypsy	223	0.16910
**CL112**	0.012	DNA.TcMar.	221	0.20410
**CL113**	0.012	DNA.TcMar.	220	0.81220
**CL114**	0.012	DNA.TcMar.Tc1	213	0.72610
**CL115**	0.011	LTR Gypsy	205	0.18590
**CL116**	0.011	Unclassified	204	0.00000
**CL117**	0.011	DNA Sola1-type	199	0.21910
**CL118**	0.011	Unclassified	195	0.00000
**CL119**	0.011	DNA.CMC.Transib	194	2.55300
**CL120**	0.010	LTR Gypsy	190	0.26460
**CL121**	0.010	DNA.CMC.Transib	189	0.10960
**CL122**	0.010	LTR Gypsy	186	0.00000

Table S3. Description of all clusters retrieved from 2144275 reads of *D. seriema* by RepeatExplorer. The satDNA families analyzed in this study are highlighted in bold red.

Cluster	Genome Proportion%	RepeatMasker Annotation	Reads number on each cluster	Outside reads with similarities %
**CL1**	**1.930**	**pBuM-2**	**41472**	**0.176**
**CL2**	1.410	RC.Helitron	30287	24.010
**CL3**	0.978	Homo6	20966	32.230
**CL4**	**0.809**	**DBC-150**	**17349**	**0.784**
**CL5**	0.730	RC.Helitron	15646	26.060
**CL6**	0.673	Non-LTR CR1	14427	5.296
**CL7**	0.607	PERI	13026	24.800
**CL8**	0.595	RC.Helitron	12761	42.540
**CL9**	0.519	LTR Gypsy	11132	2.677
**CL10**	0.504	SSS139	10798	19.010
**CL11**	0.455	Non-LTR R1	9753	0.749
**CL12**	0.450	Non-LTR R1	9650	1.907
**CL13**	0.428	LTR BEL	9170	2.345
**CL14**	0.403	rRNA	8643	0.104
**CL15**	0.401	Galileo	8605	6.392
**CL16**	0.355	LTR Gypsy	7615	2.232
**CL17**	0.345	Unclassified	7408	0.310
**CL18**	0.344	Unclassified	7385	0.163
**CL19**	0.329	RC.Helitron	7044	31.960
**CL20**	0.328	Galileo	7041	9.260
**CL21**	0.326	Non-LTR Jockey	6993	2.331
**CL22**	0.314	Non-LTR Jockey	6743	4.968
**CL23**	0.313	LTR BEL	6705	1.253
**CL24**	0.311	Unclassified	6674	1.049
**CL25**	0.311	LTR BEL	6658	6.008
**CL26**	0.272	LTR Gypsy	5826	1.442
**CL27**	**0.266**	***CDSTR138***	**5709**	**0.893**
**CL28**	0.257	Non-LTR R1	5502	3.017
**CL29**	0.253	LTR Gypsy	5433	4.013
**CL30**	0.249	LTR Gypsy	5343	1.029
**CL31**	0.238	rRNA	5094	1.924
**CL32**	0.234	LTR Copia	5022	3.146
**CL33**	0.206	rRNA	4421	3.936
**CL34**	0.176	LTR Gypsy	3781	1.878
**CL35**	0.171	LTR BEL	3663	2.266
**CL36**	0.167	LTR Gypsy	3583	5.610
**CL37**	0.160	Unclassified	3438	2.967
**CL38**	0.152	LTR Gypsy	3257	2.395
**CL39**	0.147	LTR Gypsy	3143	6.713
**CL40**	0.144	Unclassified	3082	4.121

**CL41**	0.143	LTR Copia	3076	2.503
**CL42**	0.140	LTR BEL	3011	1.328
**CL43**	0.137	LTR Gypsy	2933	0.852
**CL44**	0.136	RC.Helitron	2910	0.687
**CL45**	0.133	LTR Gypsy	2856	2.066
**CL46**	0.132	Unclassified	2830	0.177
**CL47**	0.129	LTR Gypsy	2766	3.868
**CL48**	0.129	Unclassified	2759	8.191
**CL49**	0.123	LTR Gypsy	2647	2.909
**CL50**	0.115	RC.Helitron/DNA.hAT.Pegasus	2474	0.000
**CL51**	0.115	Non-LTR I	2473	3.275
**CL52**	0.114	LTR BEL	2440	3.238
**CL53**	0.111	LTR Gypsy	2378	0.925
**CL54**	0.111	DNA.Ginger	2373	6.153
**CL55**	0.109	Unclassified	2343	0.427
**CL56**	0.107	LTR Gypsy	2289	3.102
**CL57**	0.100	LTR Gypsy	2152	2.138
**CL58**	0.098	Non-LTR Jockey	2100	0.048
**CL59**	0.096	Galileo	2061	2.232
**CL60**	0.094	LTR BEL	2023	3.114
**CL61**	0.091	LTR Gypsy	1960	2.092
**CL62**	0.090	LTR Gypsy	1922	1.769
**CL63**	0.089	RC.Helitron	1919	1.928
**CL64**	0.089	LTR Gypsy	1910	1.571
**CL65**	0.089	LTR Gypsy	1899	2.791
**CL66**	0.088	LTR Gypsy	1895	3.219
**CL67**	0.088	Non-LTR Jockey	1883	1.009
**CL68**	0.085	LTR Gypsy	1832	4.039
**CL69**	0.082	Non-LTR Jockey	1758	2.446
**CL70**	0.079	Non-LTR Jockey	1698	4.005
**CL71**	0.078	DNA.TcMar.Tc1	1665	3.363
**CL72**	0.075	LTR Gypsy	1612	1.365
**CL73**	0.074	Unclassified	1596	0.125
**CL74**	0.074	Unclassified	1586	0.568
**CL75**	0.072	Unclassified	1555	0.000
**CL76**	0.072	DNA.CMC.Transib	1545	24.140
**CL77**	0.071	LTR BEL	1530	1.046
**CL78**	0.069	LTR Gypsy	1486	2.826
**CL79**	0.067	DNA.hAT.Tip100	1435	3.902
**CL80**	0.067	DNA.TcMar.Tc1	1429	2.799
**CL81**	0.066	LTR ERVL	1411	0.071
**CL82**	0.066	Unclassified	1406	0.854
**CL83**	0.065	Non-LTR Jockey	1400	0.643
**CL84**	0.064	DNA.TcMar.Tc1	1366	2.928
**CL85**	0.062	LTR Copia	1340	0.298

**CL86**	0.061	Galileo	1311	3.051
**CL87**	0.061	LTR Copia	1306	2.374
**CL88**	0.059	Non-LTR Jockey	1263	3.959
**CL89**	0.059	Non-LTR Jockey	1261	1.983
**CL90**	0.057	LTR Gypsy	1215	2.881
**CL91**	0.056	DNA.hAT.Ac	1200	5.917
**CL92**	0.054	Unclassified	1165	0.000
**CL93**	0.054	DNA.TcMar.Tc1	1163	4.729
**CL94**	0.049	Non-LTR RTE	1043	1.534
**CL95**	0.048	LTR Gypsy	1019	0.687
**CL96**	0.045	LTR Gypsy	969	0.516
**CL97**	0.044	LTR Gypsy	953	3.043
**CL98**	0.044	Unclassified	940	0.319
**CL99**	0.044	LTR Gypsy	934	0.107
**CL100**	0.043	Unclassified	932	0.000
**CL101**	0.042	LTR Gypsy	903	0.111
**CL102**	0.042	DNA.Maverick	895	0.335
**CL103**	0.041	Unclassified	869	0.115
**CL104**	0.038	LTR Gypsy	810	2.469
**CL105**	0.037	DNA.hAT.Pegasus	800	4.625
**CL106**	0.037	Unclassified	785	0.255
**CL107**	0.035	Unclassified	749	0.000
**CL108**	0.033	LTR Gypsy	715	2.378
**CL109**	0.033	Unclassified	709	0.000
**CL110**	0.033	Non-LTR R1	709	1.975
**CL111**	0.033	LTR Copia	701	0.856
**CL112**	0.032	Unclassified	688	0.000
**CL113**	0.030	Unclassified	639	0.157
**CL114**	0.029	Unclassified	631	0.000
**CL115**	0.029	LTR Gypsy	625	3.520
**CL116**	0.029	LTR Copia	624	3.526
**CL117**	0.029	LTR Gypsy	623	3.371
**CL118**	0.029	Unclassified	620	0.000
**CL119**	0.028	Unclassified	605	0.000
**CL120**	0.028	LTR Gypsy	600	1.167
**CL121**	0.028	Non-LTR I	595	0.840
**CL122**	0.027	Unclassified	585	0.000
**CL123**	0.027	Unclassified	579	0.000
**CL124**	0.027	DNA.PIF	575	0.000
**CL125**	0.026	Unclassified	550	0.000
**CL126**	0.026	Unclassified	549	0.000
**CL127**	0.025	LTR Gypsy	541	0.000
**CL128**	0.025	Galileo	532	4.511
**CL129**	0.025	DNA.TcMar.Tc1	531	4.520
**CL130**	0.025	LTR Copia	528	0.379
**CL131**	0.024	RC.Helitron	521	0.000

**CL132**	0.024	LTR Gypsy	518	1.158
**CL133**	**0.024**	**CDSTR198**	**513**	**0.195**
**CL134**	0.024	LTR Gypsy	503	0.000
**CL135**	0.023	LTR Copia	499	1.804
**CL136**	0.023	Unclassified	493	0.000
**CL137**	0.022	Unclassified	483	0.207
**CL138**	0.022	Unclassified	480	0.000
**CL139**	0.022	Unclassified	479	0.000
**CL140**	0.022	Galileo	476	4.202
**CL141**	0.022	Unclassified	470	0.000
**CL142**	0.022	Unclassified	467	0.214
**CL143**	0.021	DNA.TcMar.Tc1	449	3.786
**CL144**	0.020	Unclassified	438	0.000
**CL145**	0.020	Unclassified	436	0.000
**CL146**	0.020	Unclassified	436	0.000
**CL147**	0.020	LTR Gypsy	434	1.152
**CL148**	0.020	Unclassified	432	0.232
**CL149**	0.020	RC.Helitron	430	3.953
**CL150**	0.020	Non-LTR L1	429	0.000
**CL151**	0.020	LTR Gypsy	426	0.000
**CL152**	0.020	Unclassified	426	0.000
**CL153**	0.020	Unclassified	424	0.236
**CL154**	0.020	Unclassified	423	0.000
**CL155**	0.020	Unclassified	420	0.000
**CL156**	0.020	Unclassified	420	0.000
**CL157**	0.020	Unclassified	420	0.000
**CL158**	0.019	Unclassified	419	0.000
**CL159**	0.019	Non-LTR Jockey	412	1.456
**CL160**	0.019	RC.Helitron	410	2.439
**CL161**	0.019	DNA.hAT.hATm	410	0.000
**CL162**	0.019	DNA.hAT.hobo	409	1.711
**CL163**	0.019	Unclassified	407	0.000
**CL164**	0.019	Unclassified	405	0.000
**CL165**	0.019	Unclassified	404	0.000
**CL166**	0.019	Unclassified	399	0.000
**CL167**	0.018	LTR BEL	397	0.000
**CL168**	0.018	LTR Gypsy	396	0.505
**CL169**	0.018	Unclassified	394	0.000
**CL170**	0.018	LTR BEL	394	1.523
**CL171**	0.018	Unclassified	392	0.000
**CL172**	0.018	Unclassified	389	0.000
**CL173**	0.018	Unclassified	387	0.000
**CL174**	0.018	DNA.CMC.Transib	387	8.010
**CL175**	0.018	Unclassified	383	0.000
**CL176**	0.018	Unclassified	381	0.000
**CL177**	0.018	Unclassified	380	0.000

**CL178**	0.018	Unclassified	379	0.000
**CL179**	0.018	Unclassified	379	0.000
**CL180**	0.017	DNA.TcMar.Tc1	374	1.337
**CL181**	0.017	Unclassified	373	0.000
**CL182**	0.017	Unclassified	371	0.000
**CL183**	0.017	Unclassified	370	0.000
**CL184**	0.017	Unclassified	369	0.000
**CL185**	0.017	DNA.TcMar.Tc1	365	1.370
**CL186**	0.017	Unclassified	365	0.000
**CL187**	0.017	Unclassified	365	0.000
**CL188**	0.017	Unclassified	365	0.000
**CL189**	0.017	DNA.hAT	365	0.000
**CL190**	0.017	DNA.CMC.Transib	364	0.000
**CL191**	0.017	Unclassified	364	0.000
**CL192**	0.017	Unclassified	364	0.000
**CL193**	0.017	Unclassified	363	0.000
**CL194**	0.017	Unclassified	358	0.000
**CL195**	0.017	LTR Copia	356	0.281
**CL196**	0.017	Unclassified	356	0.000
**CL197**	0.017	Unclassified	354	0.000
**CL198**	0.017	Unclassified	353	0.000
**CL199**	0.017	Unclassified	353	0.000
**CL200**	0.016	Unclassified	349	0.286
**CL201**	0.016	rDNA	349	0.000
**CL202**	0.016	tRNA	349	0.000
**CL203**	0.016	Unclassified	349	0.000
**CL204**	0.016	Unclassified	348	0.000
**CL205**	0.016	Unclassified	347	0.000
**CL206**	0.016	Unclassified	346	0.000
**CL207**	0.016	Unclassified	344	0.000
**CL208**	0.016	LTR BEL	343	0.000
**CL209**	0.016	Unclassified	339	0.000
**CL210**	0.016	Unclassified	337	0.000
**CL211**	0.016	Unclassified	335	0.000
**CL212**	0.015	Unclassified	332	0.000
**CL213**	0.015	Unclassified	331	0.000
**CL214**	0.015	Unclassified	328	0.000
**CL215**	0.015	Unclassified	328	0.000
**CL216**	0.015	Unclassified	326	0.000
**CL217**	0.015	LTR ERVK	326	0.000
**CL218**	0.015	Unclassified	324	0.000
**CL219**	0.015	Unclassified	323	0.000
**CL220**	0.015	Unclassified	323	0.000
**CL221**	0.015	Unclassified	319	0.000
**CL222**	0.015	Unclassified	313	0.000
**CL223**	0.015	Unclassified	312	0.000

**CL224**	0.015	Unclassified	311	0.000
**CL225**	0.015	Unclassified	311	0.000
**CL226**	0.015	Unclassified	311	0.000
**CL227**	0.014	Unclassified	308	0.000
**CL228**	0.014	Unclassified	304	0.000
**CL229**	0.014	DNA.CMC.Chapaev	304	0.000
**CL230**	0.014	Unclassified	302	0.000
**CL231**	0.014	Unclassified	300	0.000
**CL232**	0.014	Unclassified	300	0.000
**CL233**	0.014	Unclassified	299	0.000
**CL234**	0.014	LTR Gypsy	299	0.000
**CL235**	0.014	Unclassified	299	0.000
**CL236**	0.014	Unclassified	298	0.000
**CL237**	0.014	Unclassified	296	0.000
**CL238**	0.014	Non-LTR CR1	296	0.000
**CL239**	0.014	DNA.CMC.EnSpm	295	0.000
**CL240**	0.014	Unclassified	295	0.000
**CL241**	0.014	Unclassified	292	0.000
**CL242**	0.014	Unclassified	291	0.000
**CL243**	0.013	Unclassified	289	0.000
**CL244**	0.013	Non-LTR I	287	0.000
**CL245**	0.013	Unclassified	287	0.000
**CL246**	0.013	Unclassified	286	0.000
**CL247**	0.013	Unclassified	286	0.000
**CL248**	0.013	Unclassified	283	0.000
**CL249**	0.013	DNA.TcMar.Tc	282	2.482
**CL250**	0.013	Unclassified	279	0.000
**CL251**	0.013	LTR Copia	277	0.000
**CL252**	0.013	Unclassified	276	0.000
**CL253**	0.013	Unclassified	274	0.000
**CL254**	0.013	Unclassified	274	0.000
**CL255**	0.013	Unclassified	273	0.000
**CL256**	0.013	Unclassified	273	0.000
**CL257**	0.013	Unclassified	273	0.000
**CL258**	0.013	Unclassified	273	0.000
**CL259**	0.013	DNA.Maverick	271	1.107
**CL260**	0.013	Unclassified	268	0.000
**CL261**	0.013	DNA.PiggyBac	268	0.746
**CL262**	0.013	Unclassified	267	0.000
**CL263**	0.012	Unclassified	265	0.000
**CL264**	0.012	Unclassified	264	0.000
**CL265**	0.012	Unclassified	263	0.000
**CL266**	0.012	Unclassified	263	0.000
**CL267**	0.012	Unclassified	262	0.000
**CL268**	0.012	Unclassified	261	0.000
**CL269**	0.012	Unclassified	259	0.000

**CL270**	0.012	Unclassified	259	0.000
**CL271**	0.012	Unclassified	257	0.000
**CL272**	0.012	LTR Gypsy	257	0.000
**CL273**	0.012	Unclassified	255	0.000
**CL274**	0.012	Unclassified	254	0.000
**CL275**	0.012	LTR ERVK	254	0.000
**CL276**	0.012	Unclassified	253	0.000
**CL277**	0.012	Unclassified	251	0.000
**CL278**	0.012	Unclassified	251	0.000
**CL279**	0.012	Unclassified	250	0.000
**CL280**	0.012	Unclassified	250	0.000
**CL281**	0.012	Unclassified	248	0.000
**CL282**	0.011	Unclassified	247	0.000
**CL283**	0.011	Unclassified	246	0.000
**CL284**	0.011	Unclassified	245	0.000
**CL285**	0.011	Unclassified	245	0.000
**CL286**	0.011	Unclassified	244	0.000
**CL287**	0.011	Unclassified	243	0.000
**CL288**	0.011	Unclassified	243	0.411
**CL289**	0.011	Unclassified	241	0.000
**CL290**	0.011	Unclassified	240	0.000
**CL291**	0.011	Unclassified	238	0.000
**CL292**	0.011	Unclassified	238	0.000
**CL293**	0.011	LTR Copia	238	0.840
**CL294**	0.011	Unclassified	237	0.000
**CL295**	0.011	Unclassified	237	0.000
**CL296**	0.011	Unclassified	236	0.000
**CL297**	0.011	Unclassified	235	0.000
**CL298**	0.011	Unclassified	235	0.000
**CL299**	0.011	Unclassified	234	0.000
**CL300**	0.011	Unclassified	234	0.000
**CL301**	0.011	Unclassified	234	0.000
**CL302**	0.011	Unclassified	233	0.000
**CL303**	0.011	Unclassified	233	0.000
**CL304**	0.011	Unclassified	231	0.000
**CL305**	0.011	Unclassified	230	0.000
**CL306**	0.011	Unclassified	229	0.000
**CL307**	0.011	Unclassified	227	0.000
**CL308**	0.011	DNA.PIF.ISL2EU	226	0.000
**CL309**	0.011	Unclassified	226	0.000
**CL310**	0.011	Unclassified	225	0.000
**CL311**	0.011	Unclassified	225	0.000
**CL312**	0.010	Unclassified	224	0.000
**CL313**	0.010	Unclassified	224	0.000
**CL314**	0.010	Non-LTR Penelope	224	0.000
**CL315**	0.010	Unclassified	223	0.000

**CL316**	0.010	Unclassified	222	0.000
**CL317**	0.010	LTR Gypsy	221	0.000
**CL318**	0.010	Unclassified	221	0.000
**CL319**	0.010	Unclassified	220	0.000
**CL320**	0.010	Unclassified	219	0.000
**CL321**	0.010	Non-LTR CR1	219	0.000
**CL322**	0.010	Unclassified	219	0.000
**CL323**	0.010	Unclassified	218	0.000
**CL324**	0.010	Unclassified	218	0.000
**CL325**	0.010	Unclassified	218	0.459
**CL326**	0.010	Unclassified	218	0.000
**CL327**	0.010	Unclassified	216	0.000
**CL328**	0.010	Unclassified	214	0.000

Table S4. Description of all clusters retrieved from 2174346 reads of *D. mojavensis* by RepeatExplorer. The satDNA families analyzed in this study are highlighted in bold red.

Cluster	Genome Proportion %	RepeatMasker Annotation	Reads number on each cluster	Outside reads with similarity %
**CL1**	**1.630**	***CDSTR130***	**35395**	**0.494**
**CL2**	**0.861**	***pBuM***	**18724**	**0.417**
**CL3**	0.625	RC.Helitron	13585	41.430
**CL4**	0.616	Homo6	13402	45.750
**CL5**	0.614	RC.Helitron	13345	42.750
**CL6**	0.482	Homo6	10483	33.900
**CL7**	0.475	RC.Helitron	10323	42.020
**CL8**	0.464	Non-LTR Jockey	10096	0.366
**CL9**	0.435	RC.Helitron	9461	21.380
**CL10**	0.429	Galileo	9320	2.157
**CL11**	0.420	Non-LTR R1	9132	1.818
**CL12**	0.340	RC.Helitron	7382	31.140
**CL13**	0.323	Unclassified	7027	0.896
**CL14**	0.299	RC.Helitron	6496	3.079
**CL15**	0.298	Non-LTR R1	6480	0.972
**CL16**	0.281	LTR BEL	6119	0.082
**CL17**	0.239	Unclassified	5196	0.019
**CL18**	0.227	Non-LTR R1	4944	3.621
**CL19**	0.227	Non-LTR CR1	4935	2.411
**CL20**	0.208	Non-LTR Jockey	4515	0.975
**CL21**	0.200	rRNA	4355	0.987
**CL22**	0.194	LTR BEL	4216	0.427
**CL23**	0.194	Non-LTR R1	4208	0.048
**CL24**	0.188	PERI	4096	29.050
**CL25**	0.178	Non-LTR Jockey	3860	0.130
**CL26**	0.177	LTR Gypsy	3856	0.778
**CL27**	0.175	Non-LTR Jockey	3814	0.000
**CL28**	0.166	Non-LTR Jockey /TART	3605	3.523
**CL29**	0.166	rRNA	3601	1.000
**CL30**	0.157	LTR Gypsy	3420	1.637
**CL31**	0.157	LTR Gypsy	3409	0.264
**CL32**	0.156	LTR BEL	3392	0.884
**CL33**	0.138	LTR Gypsy	3007	0.200
**CL34**	0.137	LTR Gypsy	2978	0.000
**CL35**	0.135	RC.Helitron	2925	9.983
**CL36**	0.132	LTR Gypsy	2867	1.604
**CL37**	0.111	RC.Helitron	2413	2.445
**CL38**	0.110	DNA.hAT.Ac	2399	0.125
**CL39**	0.107	Rep2-DF	2337	2.696

**CL40**	0.106	LTR Copia	2302	0.174
**CL41**	0.097	DNA.TcMar.Tc1	2103	3.281
**CL42**	0.094	LTR BEL	2045	1.125
**CL43**	0.093	DNA.TcMar.Tc1	2032	1.280
**CL44**	0.077	DNA.TcMar.Tc1	1681	0.833
**CL45**	0.072	LTR BEL	1570	3.694
**CL46**	0.072	Unclassified	1559	1.668
**CL47**	0.071	DNA.CMC.Transib	1540	0.779
**CL48**	0.069	LTR Gypsy	1492	0.335
**CL49**	0.067	LTR Gypsy	1448	0.691
**CL50**	0.063	Unclassified	1379	2.321
**CL51**	0.062	DNA.hAT.Tip100	1360	2.426
**CL52**	0.062	Unclassified	1358	0.074
**CL53**	0.059	Unclassified	1284	0.000
**CL54**	0.054	LTR Gypsy	1177	1.359
**CL55**	0.053	Non-LTR R1	1164	0.172
**CL56**	0.052	Non-LTR Jockey	1126	0.178
**CL57**	0.051	Unclassifeid	1105	0.000
**CL58**	0.050	LTR BEL	1093	0.366
**CL59**	0.049	Galileo	1066	1.876
**CL60**	0.048	LTR BEL	1045	0.766
**CL61**	0.041	Non-LTR Jockey	883	1.133
**CL62**	0.040	LTR Gypsy	870	0.575
**CL63**	0.040	LTR Gypsy	869	0.000
**CL64**	0.037	DNA.TcMar.Mariner	804	0.746
**CL65**	0.036	DNA.TcMar.Tc1	780	0.000
**CL66**	0.034	DNA.TA-1	731	1.231
**CL67**	0.033	DNA-1_RPr	724	0.000
**CL68**	0.030	LTR Gypsy	661	1.210
**CL69**	0.030	RC.Helitron	657	0.457
**CL70**	0.029	Non-LTR Jockey	627	0.478
**CL71**	0.026	LTR BEL	565	1.593
**CL72**	0.026	LTR Gypsy	562	0.000
**CL73**	0.025	Unclassified	537	0.000
**CL74**	0.024	LTR Gypsy	527	0.000
**CL75**	0.024	Non-LTR Jockey	518	1.158
**CL76**	0.023	DNA.TcMar.Tc1	502	0.398
**CL77**	0.023	LTR ERV1	496	0.202
**CL78**	0.022	DNA.TcMar.Tc1	482	5.394
**CL79**	0.022	DNA.TcMar.Tc1	472	0.424
**CL80**	0.021	rDNA	454	0.000
**CL81**	0.020	LTR BEL	442	0.226
**CL82**	0.020	DNA.piggyBac	441	1.134
**CL83**	0.020	DNA.TcMar.	438	1.598
**CL84**	0.019	Non-LTR Jockey	417	0.000
**CL85**	0.018	Non-LTR CR1	381	7.612

**CL86**	0.017	Non-LTR Jockey	367	0.273
**CL87**	0.017	Non-LTR Jockey	362	0.276
**CL88**	0.016	LTR Gypsy	354	1.412
**CL89**	0.014	Unclassifeid	310	18.710
**CL90**	0.014	LINE	298	0.000
**CL91**	0.013	DNA.hAT.Ac	293	0.341
**CL92**	0.013	DNA.hAT.Pegasus	289	2.422
**CL93**	0.013	DNA.hAT.hobo	288	3.819
**CL94**	0.013	LTR Gypsy	282	0.000
**CL95**	0.013	DNA.TcMar.Tc1	278	2.158
**CL96**	0.013	LTR Gypsy	273	0.000
**CL97**	0.012	Unclassifeid	256	0.000
**CL98**	0.012	Unclassifeid	255	0.000
**CL99**	0.011	Unclassifeid	248	0.000
**CL100**	0.011	Non-LTR Jockey	244	0.410
**CL101**	0.011	LTR BEL	243	0.000
**CL102**	0.011	DNA.PIF.Harbinger	242	1.653
**CL103**	0.011	DNA.TcMar.Tc1	237	1.266
**CL104**	0.011	LTR Gypsy	235	0.425
**CL105**	0.010	DNA.CMC.Transib	226	0.443
**CL106**	0.010	DNA.hAT.hobo	224	3.125
**CL107**	0.010	DNA.hAT.Pegasus	217	3.687
**CL108**	0.010	Unclassified	217	0.000
**CL109**	0.010	DNA.TcMar.Tc1	217	0.461

Table S5. Description of the ten most abundant clusters of the *D. melanogaster* genome identified by RepeatExplorer. The satDNA families with monomer lengths smaller than 50 bp are highlighted in bold.

Cluster	Sequence Annotation	Monomer size (bp)	Genome proportion %	Number of reads on each cluster
**1**	**TGTTATTCTA**	**10**	**1.750**	**86909**
**2**	**AGAGA**	**5**	**0.715**	**35548**
**3**	*1.688*	360	0.551	27393
**4**	**AGACA**	**5**	**0.484**	**24099**
**5**	*1.688*	360	0.453	22558
**6**	*1.688*	360	0.437	21759
**7**	***dodeca***	**11-12**	**0.380**	**18912**
**8**	*1.688*	360	0.340	16906
**9**	*Responder*	240	0.323	16044
**10**	LTR-Gypsy	-	0.293	14577

Table S6. Main features of 37 *CDSTR198* arrays located on euchromatic regions and their chromosome location according to GenomeBrowser analysis.

Contig	Array Size (bp)	Copy number	Scaffold	Chromosome
713	300	1,51	1	2
714	521	2,63	1	2
834	643	3,24	1	2
894	563	2,84	1_2	2
1013	585	2,95	1_2	2
1263	908	4,58	2	2
1281	462	2,33	2	2
1732	557	2,81	1_2	2
2138	348	1,75	6	3
2239	912	4,6	6	3
2290	814	4,1	6	3
3200	1044	5,27	11	4
4011	884	4,46	13	5
4217	627	3,16	14	5
4994	243	1,22	22	4
5012	530	2,67	22	4
5199	608	3,07	18_1	3
5294	450	2,27	18_1	3
5693	741	3,74	18_2	3
6645	233	1,17	28	X
6973	804	4,06	22	4
8355	287	1,45	40	4
8688	625	3,15	41	5
8952	405	2,04	31	4
9897	499	2,52	52	2
12072	243	1,22	6	3
12476	659	3,33	83	3
13070	678	3,42	24	3
13095	680	3,43	24	3
13237	652	3,29	68	5
14801	521	2,63	121	5
15065	692	3,49	123	4
16146	616	3,11	174	Unmapped
18240	404	2,04	111	5
20000	607	3,06	18	3
27251	322	1,62	2	2
31812	196	0,99	2	2

Table S7. List of genes associated with *CDSTR198* arrays and their relative positions in relation to *CDSTR198*.

**Contig**	**Array Size (bp)**	**Position of CDSTR198 relative to gene**	**Distance of CDSTR198 array to gene**	**Gene annotation**	**D. melanogaster orthologous**	**Scaffold/ chromosome**
714	521	Intron	-	EVM prediction scaffold1.763	FBpp0082178	1/2
894	563	Intron	-	EVM prediction scaffold 1.1077	FBpp0111784	1/2
1013	585	Intron	-	EVM prediction scaffold1.1420	FBpp0303067	1/2
2138	348	Intron	-	EVM prediction scaffold6.61	FBpp0080953	6/3
4011	884	5’	1.4 kb	EVM prediction scaffold13.141	FBpp0167799	13/5
4217	627	5’	750 bp	FBpp0170282	-	14/5
5012	530	5’	500 bp	EVM prediction scaffold22.170	-	22/4
5199	608	5’	4 kb	Gene.g4503.tl	-	18/3
6645	233	5’	3 kb	EVM prediction scaffold28.31	FBpp0073879	28/X
6973	804	5’	800 bp	EVM prediction scaffold22.28	FBpp0072761	22/4
8355	287	5’	1.9 kb	EVM prediction scaffold40.3	FBpp0072462	40/4
8688	625	Intron	-	EVM prediction scaffold41.61	FBpp0112268	41/5
8952	405	5’	500 bp	EVM prediction scaffold31.206	FBpp0079016	31/4
13070	678	5’	800 bp	EVM prediction scaffold24_1.4	-	24/3
13095	680	5’	300 bp	SNAPPosition:scaffold24_preditec gene_268763..270446	-*	24/3
14801	521	5’	3,9 kb	EVM prediction scaffold121.6	FBpp0074830	121/5

*This predicted gene has no orthologous on *D. melanogaster* or *D. mojavensis* according to *Drosophila buzzatii* Genome Project (http://dbuz.uab.cat; Guillen et al. 2015)
